# Quantifying shifts in natural selection on codon usage between protein regions: a population genetics approach

**DOI:** 10.1186/s12864-022-08635-0

**Published:** 2022-05-30

**Authors:** Alexander L. Cope, Michael A. Gilchrist

**Affiliations:** 1 Genome Science and Technology, University of Tennessee, Knoxville, United States; 2 Current Address: Department of Genetics, Rutgers University, Piscataway, United States; 3 National Institute for Mathematical and Biological Synthesis, Knoxville, TN United States; 4 Department of Ecology and Evolutionary Biology, University of Tennessee, Knoxville, United States

**Keywords:** Codon usage bias, Protein folding, Protein secondary structure, Population genetics

## Abstract

**Background:**

Codon usage bias (CUB), the non-uniform usage of synonymous codons, occurs across all domains of life. Adaptive CUB is hypothesized to result from various selective pressures, including selection for efficient ribosome elongation, accurate translation, mRNA secondary structure, and/or protein folding. Given the critical link between protein folding and protein function, numerous studies have analyzed the relationship between codon usage and protein structure. The results from these studies have often been contradictory, likely reflecting the differing methods used for measuring codon usage and the failure to appropriately control for confounding factors, such as differences in amino acid usage between protein structures and changes in the frequency of different structures with gene expression.

**Results:**

Here we take an explicit population genetics approach to quantify codon-specific shifts in natural selection related to protein structure in *S. cerevisiae* and *E. coli*. Unlike other metrics of codon usage, our approach explicitly separates the effects of natural selection, scaled by gene expression, and mutation bias while naturally accounting for a region’s amino acid usage. Bayesian model comparisons suggest selection on codon usage varies only slightly between helix, sheet, and coil secondary structures and, similarly, between structured and intrinsically-disordered regions. Similarly, in contrast to prevous findings, we find selection on codon usage only varies slightly at the termini of helices in *E. coli*. Using simulated data, we show this previous work indicating “non-optimal” codons are enriched at the beginning of helices in *S. cerevisiae* was due to failure to control for various confounding factors (e.g. amino acid biases, gene expression, etc.), and rather than selection to modulate cotranslational folding.

**Conclusions:**

Our results reveal a weak relationship between codon usage and protein structure, indicating that differences in selection on codon usage between structures are slight. In addition to the magnitude of differences in selection between protein structures being slight, the observed shifts appear to be idiosyncratic and largely codon-specific rather than systematic reversals in the nature of selection. Overall, our work demonstrates the statistical power and benefits of studying selective shifts on codon usage or other genomic features from an explicitly evolutionary approach. Limitations of this approach and future potential research avenues are discussed.

**Supplementary Information:**

The online version contains supplementary material available at (10.1186/s12864-022-08635-0).

## Background

Patterns of codon usage bias (CUB), or the non-uniform usage of synonymous codons, vary both within and across species [1–3]. Although non-adaptive evolutionary forces (e.g. mutation biases, GC-biased gene conversion) are well-known to shape codon usage patterns, natural selection also plays a significant role. The correlation between codon frequency and tRNA abundances and the bias towards more efficient codons in highly expressed genes suggests selection against translation inefficiency is a major factor shaping genome-wide codon patterns [4–6]. Codon usage is also known to vary within a gene, which is hypothesized to reflect various other forces of selection [7]. For example, CUB is thought to be shaped by selection for translation accuracy, such as to reduce missense errors at functionally-important sites and the frequency of ribosomal drop-off along a transcript, both of which can result in non-functional proteins [8–11]. Intragenic variation in synonymous codon usage has also been proposed to be shaped by selection to prevent ribosomal queuing [12] and selection to avoid mRNA secondary structure near the ends of mRNA transcripts [13–15]. Furthermore, synonymous codon usage has been hypothesized to tune time-sensitive processes realted to protein folding and secretion [16, 17].

Although adaptive CUB is thought to be largely driven by selection for translation efficiency, research indicates potential selective advantages of inefficient codons (“non-optimal” or “rare” codons) at certain sites within a protein [18]. Given that codon usage patterns are strongly shaped by amino acid biases, mutation biases, and gene expression, it is important for researchers investigating possible adaptive codon usage patterns to ensure that these patterns cannot be explained by non-adaptive factors. Gould and Lewontin [19] highlighted the bias of biologists towards adaptationist storytelling, arguing that non-adaptive evolutionary forces (e.g. genetic drift) and other constraints (e.g. development) should be considered before attributing a trait or behavior to adaptive evolution. With the ushering in of the genomic-era, similar arguments have been made about the importance of testing hypotheses related to selection on and adaptation of genomic features in the context of the evolutionary null, i.e. the expectation in the absence of selection [20, 21]. Here, we will use a population genetics based model of coding sequence evolution with clearly defined null formulations to investigate variation in selection on codon usage related to protein structure.

It is generally accepted that misfolded proteins have impaired function, can aggregate within the cell, and possibly disrupt key cellular processes [10, 22, 23]. Missense errors are hypothesized to increase the frequency of protein misfolding; thus, regions important for the protein folding are expected to be under stronger selection for translationally-accurate codons [10]. In addition to the effects of missense errors, codon usage can modulate protein folding via changes in the elongation rates at key steps during cotranslational folding [16]. Empirical evidence indicate changes to elongation rates via synonymous codon usage can alter cotranslational protein folding in organisms ranging from bacteria to multicellular eukaryotes [24–29]. Synonymous changes to codon usage are known to impact cellular fitness and have been implicated in human diseases through altered protein folding [30, 31]. Understanding the role of codon usage in protein folding also has biotechnological significance, as a recombinant protein is often expressed in an organism with a drastically different CUB, potentially perturbing cotranslational folding [24].

Given that protein secondary structures generally differ in physicochemical properties and ability to cotranslationally fold, researchers have hypothesized that different structures will exhibit different patterns of CUB [17, 18, 32]. Numerous studies have examined the relationship between CUB and protein secondary structure, but a general relationship, if any, remains unclear [17, 32–37]. Other analyses of CUB at higher levels of protein structure have reached different conclusions about the relationship between codon usage and protein structure [32, 37–40].

Two probable causes for the inconsistencies across studies are (1) the different approaches for quantifying CUB across and within genes and (2) the different ways in which a gene or region is defined as being under selection at the codon usage level. Various heuristic approaches have been developed to identify selectively-favored codons and estimate the degree of codon adaptation of a gene. For example, the Codon Adaptation Index (CAI) relies on a set of a priori identified reference genes that are thought to be under strong selection for codon usage (i.e. highly-expressed genes) in order to estimate individual codons relative adaptiveness to its synonyms [41]. In contrast, the tRNA Adaptation Index (tAI) estimates absolute codon weights (i.e. weights are not scaled relative to synoyms) based on the abundance or, more frequently, gene copy number of the tRNA with the correct anticodon, while also penalizing for wobble between the codon and anticodon [42, 43]. Neither of these commonly-used metrics considers the impact of mutation biases or how translational selection on codon usage scales with gene expression, leading to issues with identifying which codons are selectively-favored and estimating the level of codon adaptation of a gene [6, 44–47]. Metrics such as CAI that rely on codon frequencies (either genome-wide or in a reference set) can misidentify the selectively-favored codon for an amino acid if selection is weak relative to mutation bias and genetic drift, such that the actual selectively-favored codon is not the most frequently used codon, even in highly expressed genes [6, 44, 45]. Aside from leading to misidentification of the selectively-favored codon, this could lead to an underestimation of a gene’s degree of codon adaptation. In contrast, if mutation and translational selection favor the same codons, it seems likely that metrics like tAI will overestimate the codon adaptation of a gene, as it is unable to distinguish between selection and mutation bias.

Other problems emerge when attempting to use these metrics to infer differences in the nature of selection within genes. Metrics like tAI that do not consider relative differences between synonymous codons, but absolute differences across all codons, are particularly prone to amino acid biases when comparing codon usage patterns [18, 48]. While many studies often delineate codons into subsets of “optimal” and “non-optimal” codons, the criterion for classification varies between studies [17, 49, 50]. Indeed, determining codon optimality using tRNA-based metrics has led to the odd situation where all synonyms for an amino acid are classified as optimal or non-optimal [17]. Additionally, the selectively-favored codon may vary depending on the selective pressure, e.g. the most efficient codon may not be the most accurate codon, thus broad terms such as “optimal” lack context [51, 52]. Due to the variation in codon preference across selective pressures, we prefer the phrase “most selectively-favored codon.” As the strength of selection on codon usage can vary across amino acids, statisitcal comparisons of codon usage metrics that consider relative differences between synonymous codons across protein regions (e.g. protein structures, signal peptides) can lead to misleading conclusions about the nature of selection on codon usage if these regions are biased towards certain amino acids. For example, if a protein region is biased towards amino acids for which selection on synonymous codon usage is weak (relative to mutation bias and genetic drift), then comparing the mean CAI between theses regions may incorrectly indicate that the nature or strength of selection on codon usage differs within these protein regions [48]. Although other studies have attempted to control for factors like amino acid biases or gene expression when studying the relationship between adaptive codon usage and protein structure, their approaches are, in addition to the codon usage metric used, *ad-hoc* in nature [17, 38].

Recent work has relied on comparative approaches to examine the functional relationship between codon usage and protein structure, recognizing that purifying selection would lead to conserved codon usage patterns [17, 37, 40], although much of this work does not explicitly model evolutionary processes (selection, mutation, drift, etc.) Alternative to species-based comparative approaches are single-genome population genetics approaches which explicitly attempt to model such evolutionary processes. Single-genome population genetics based approaches have been used in various context to examine selection on codon usage [4, 6, 11, 44]. One particularly powerful population genetics approach is the Ribosomal Overhead Cost version of Stochastic Evolutionary Model of Protein Production Rates (ROC-SEMPPR), which is able to separate out the effects of mutation and selection on codon usage by accounting for the natural variation in intergenic gene expression [6, 44, 45]. Unlike many approaches which either average codon usage over regions using heuristic metrics or delineate codons categorically as either optimal or non-optimal, ROC-SEMPPR provides quantitative, codon-specific estimates of mutation bias and natural selection. More specifically, the estimates of the model parameter *Δη* for each codon from ROC-SEMPPR reflect the population genetics parameter *sN*_*e*_ – the selection coefficient of a codon times the effective population size – in a gene of average expression.

ROC-SEMPPR was originally developed for estimating selection and mutation biases based on genome-wide codon frequencies, but recent work has used ROC-SEMPPR to investigate both intragenic and intragenomic differences in codon usage patterns [48, 53]. ROC-SEMPPR is implemented in a Bayesian framework [44, 54], allowing for model comparisons using Deviance Information or similar criteria. As a proof of principle, we tested for differences in selection on codon usage related to protein secondary structures and intrinsically-disordered regions (IDRs) in *S. cerevisiae* and *E. coli*, two common model organisms for studying CUB. Although model comparisons indicate selection on codon usage differs across protein structures in both species, these differences are relatively minor quantitative differences rather than large, systematic reversals in the directon or nature of selection on codon usage between protein structures. In other words, for both *S. cerevisiae* and *E. coli*, natural selection on codon usage is largely consistent across protein structures, with differences in selection related to different categories of protein structures likely being rare, weak, or both. This highlights a key point that was sometimes missing from previous analyses: although differences in codon usage across protein structures may be statistically significant and even reflect selective differences (assuming the proper controls are used), these effects are overall very small. Based on our results, claims that certain structures preferentially use “non-optimal”, “rare”, or “slow” codons are overstated [36, 39]. Quantitative shifts in selection are more consistent with claims that some codons are enriched in certain protein structures relative to others (again, assuming the proper controls are used).

Similar to the differences between protein secondary structures, we find evidence for slight shifts in selection between the termini and core of secondary structures, but only in a few scenarios. More importantly, we show that a previously detected enrichment of slow translating codons near the start of helices, which was proposed to be due to selection to assist in cotranslational folding [17], was the result of biases in amino acid usage and/or failing to control for the effects of gene expression. Overall, this work demonstrates the power of population genetics approaches for testing hypotheses related to intragenic differences in selection on codon usage.

## Results

### Validation of method

Previous work has made claims regarding qualitative differences in the nature of selection on codon usage related to protein structure and attributed these changes to systematic reversals in the nature of selection from rapid to slow elongation, or relaxation of selection against translation errors [36, 38, 39]. Using a simulated dataset based on the empirically-determined helices and coils from *S. cerevisiae* (1,097 genes, the smallest dataset we have between the two species), we tested for qualitative differences in selection between protein regions in a systematic manner by reversing the directionality of selection at varying frequencies. We note that these simulated sequences have the same amino acid sequences as the empirically-determined secondary strucutres, but the codon usage is determined by the provided parameters (see Methods for details). Briefly, the Uniform Selection Regions were assumed to be evolving entirely under the same selective pressure, i.e. the selection coefficients *Δη* of a codon did not change within or across these regions. In contrast, a percentage of amino acid sites in the Heterogeneous Selection Regions were randomly chosen to be evolving under the opposite selective pressure, i.e. the selection coefficients *Δη* of these codons were the opposite (i.e. multiplied by -1) of the *Δη* used in the Uniform Selection Regions. The remaining amino acid sites in the Heterogeneous Selection Regions were simulated using the same selection coefficients as in the Uniform Selection Regions. To help clarify the purpose of these simulations, this could represent the case when selection on codon usage in Uniform Selection Regions only acts to reduce translation inefficiency, while selection on codon usage in Heterogeneous Selection Regions acts to reduce inefficiency at some amino acid sites and increase inefficiency at other sites. This example broadly reflects the hypothesis that selection on codon usage qualitatively varies between protein structures to assist some structures with cotranslational folding.

When comparing the selection coefficients *Δη* estimated from the Uniform Selection Regions and the Heterogeneous Selection Regions, we clearly see that all Deming regression slopes *β* are less than 1 (Additional File 1, Fig. S1). Unsurprisingly, when 100% of sites in the Heterogenous Selection Regions are evolving under the opposing selective pressure, *Δη* is negatively-correlated and falls along the *y*=−*x* line, consistent with expectations (Additional File 1, Fig. S1A). This indicates that having sites within the Heterogenous Selection Regions evolving under opposing selective pressures (e.g. selection for and against inefficiency) reduces the selection coefficients *Δη* relative to the *Δη* estimated from the Uniform Selection Regions. These results indicate that *Δη* is a weighted average of the various selective forces shaping coding sequence evolution within a region. Insight into ROC-SEMPPR’s behavior can be gained by observing the effects of having 50% of the codons evolving under the opposite selective pressure from the remaining codons (Additional File 1, Fig. S1B). In this case, the mean *Δη* for every codon in the Hetergogenous Selection Region is 0, reflecting that ROC-SEMPPR is unable to identify the selectively-favored codon in this region and leading to a flat line (i.e Deming regression slopes *β*=0) when comparing selection estimates *Δη* between the two regions. This model behavior is expected because ROC-SEMPPR is correctling estimating the average selection coefficient of a codon within these regions. Essentially, the opposite selective pressures in this region cancel out when estimating the average selection coefficient, making it appear as if no codon is favored over its synonyms. Even when only 1% of sites were evolving under the opposing selective pressure in the Heterogeneous Selection Regions, we were able to detect a significant downward bias in *Δη* using Deming regression slopes *β* (Additional File 1, Fig. S1C–D). In addition, many *Δη* estimates show downward selective shifts (defined conservatively as when the 95% posterior probability intervals of the estimates fail to overlap, see Methods) in the Heterogeneous Selection Regions relative to the Uniform Selective Regions, also as expected. We emphasize this analysis is performed on a single simulated dataset of ∼ 1,100 genes, the smallest out of all datasets in terms of number of genes represented. Using the smallest dataset gives us a sense of the limits of our statistical power, but this should not be considered a formal power analysis.

As noted elsewhere, the *Δη* value of a codon is equal to *sN*_*e*_ value for that codon relative to the most selectively favored codon of an amino acid when encoded in a gene with average expression, i.e. *ϕ*=1. We were able to detect overall selective differences between two regions, even if only 1% of sites in one of the regions was shaped by a different selective pressure (Additional File 1, S1). Our simulated results likely represent an approximate lower bound on the number of sites under differing selective pressures necessary to detect systematic differences in natural selection between protein structures using ROC-SEMPPR. We emphasize that this test only considers the case when the nature or directionality of selection on codon usage varies frequently within a region. In this case, the Deming regression slope *β* is expected to be significantly different from 1 when comparing selection coefficients *Δη* between regions. Similarly, consistent relaxation of selection on codon usage is expected to result in *β* significantly deviating from 1. Biological examples of this include the hypothesized relaxation of selection against missense errors at sites that are less functionally-important to the protein [8] or are less likely to lead to misfolding [10, 38], and relaxed selection against ribosome drop-off at the 5’-ends of transcripts [55, 56]. In contrast, more idiosyncratic changes in selection on codon usage would not be expected to change *β*, but would manifest as shifts in the *Δη* of individual codons between regions. These shifts in *Δη* need not be in the same direction due to the various selective pressures that can act on synonymous codon usage, such as translation efficiency, translation accuracy, and mRNA secondary structure. Importantly, the various selective pressures do not necessarily favor the same codon [51, 52]. In this case, shifts in *Δη* are expected to reflect the dominant selective pressure, such that *Δη* reflects the most selectively-favored codon, with opposing selective pressures weakening this shift.

To ensure that comparing model fits with the Deviance Information Criterion (DIC) would not always result in overfit or overparameterized models being favored, we used a simulated dataset for which the nature and strength of natural selection was the same across helices, sheets, coils, structured regions, and intrinsically-disordered regions (IDRs), i.e. they were simulated using the same selection coefficients *Δη*. As expected, a model fit assuming no differences in selection between the secondary structures was 85 DIC units better than model assuming selection varied between the three secondary structures. Using this same simulated dataset, we observed only two codon-specific quantitative shifts when comparing *Δη* 120 parameter estimates across three secondary structures which is consistent with an expected false positive error rate of 0.05 (*p*=0.98 for one tail exact binomial test that the false positive error rate is greater than 0.05 with *n*=120 total comparisons between three sets of parameters with 40 parameters in each, or one tail binomial test for short; Additional File 1, Fig. S2A – C). Similarly, we observe only one codon-specific quantitative shift when comparing *Δη* estimates for simulated structured and intrinsically-disordered regions (*p*=0.60 for one tail binomial test with *n*=40 comparisons; Additional File 1, Fig. S2D).

### Selection on codon usage varies between protein secondary structures

Based on predicted secondary structures from PsiPred [57] in *S. cerevisiae*, we found that the best supported model allowed selection on codon usage to differ across helices, sheets, and coils (Table 1, Model Y_1_). Model Y_1_ is 68 DIC units better than the next best model assuming no difference in selection between helices and sheets (Y_2_), and 621 DIC units better than the null model assuming no difference across secondary structures. We obtained similar results when using empirically-determined secondary structures, but the best two models were ambiguous as to whether selection differed between helices and sheets (Additional File 1, Table S1 Y_2_ vs. Y_1_, *Δ*DIC≈1). Similar results using predicted secondary structures from PsiPred were obtained for *E. coli*. The best overall model E_1_ allowed for selection to differ across helices, sheets, and coils, with a 61 DIC unit improvement over the model assuming no differences between helices and sheets (E_2_) and 471 DIC unit improvement over the null model (E_0_). Unlike *S. cerevisiae*, similar model fits in *E. coli* using empirical data clearly favored E_1_ over the next best model E_2_ (Additional File 1, Table S2, E_2_ vs. E_1_, *Δ*DIC = 20).

**Table 1:** Comparison of model fits examining variation in codon usage between predicted protein secondary structures. The null model (Y_0_) assumes no differences in selection on codon usage between secondary structures). H: helix. E: sheet. C: coil. *Δ*DIC=DIC_*i*_−DIC_Best_

		Groupings			
Species	Model	I	II	III	*Δ*DIC
	Y_1_	H	E	C	0
	Y_2_	HE	–	C	68
*S. cerevisiae*	Y_3_	H	EC	–	285
	Y_4_	HC	E	–	425
	Y_0_	HEC	–	–	621
	E_1_	H	E	C	0
	E_2_	HE	–	C	61
*E. coli*	E_3_	H	EC	–	251
	E_4_	HC	E	–	358
	E_0_	HEC	–	–	471

### Comparing selection *Δη* on codon usage between secondary structures

**Fig. 1:**
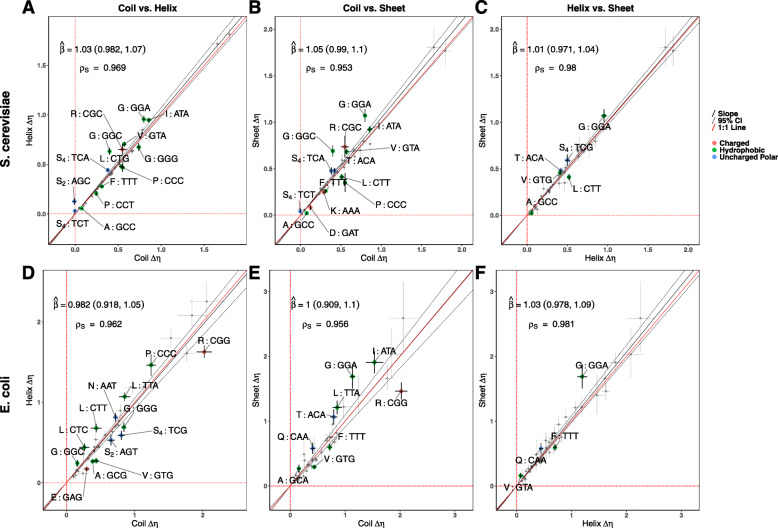
Comparison of selection estimates *Δη* between different protein secondary structures. values are scaled relative to the genome-wide most selectively-favored codon. Points represent values for each codon, while error bars represent the 95% posterior probability intervals. Codons showing significant selective shifts are colored by amino acid property. Negative values indicate a change in the selectively-favored codon relative to the genome-wide most selectively-favored codon. The Deming regression slope *β* and 95% confidence intervals (noted in parentheses) are represented by solid and dashed black lines, respectively. *ρ*_*S*_ indicates the Spearman rank correlation between the *Δη* of the two regions. **(A,D)** Coils vs. Helices. **(B,E)** Coils vs. Sheets. **(C,F)** Helices vs. Sheets

Model fits using predicted secondary structures from PsiPred [57] indicate selective shifts on codon usage across protein secondary structures. Comparing selection estimates *Δη* (based on predicted secondary structures) between protein secondary structures with a Deming regression revealed no significant differences between any of the three secondary structures (Fig. 1) in either species. Combined with our simulation work, this suggests the frequency of qualitative selective shifts between any of the three secondary structures is rare, i.e. likely < 1*%* of sites.

**Fig. 2:**
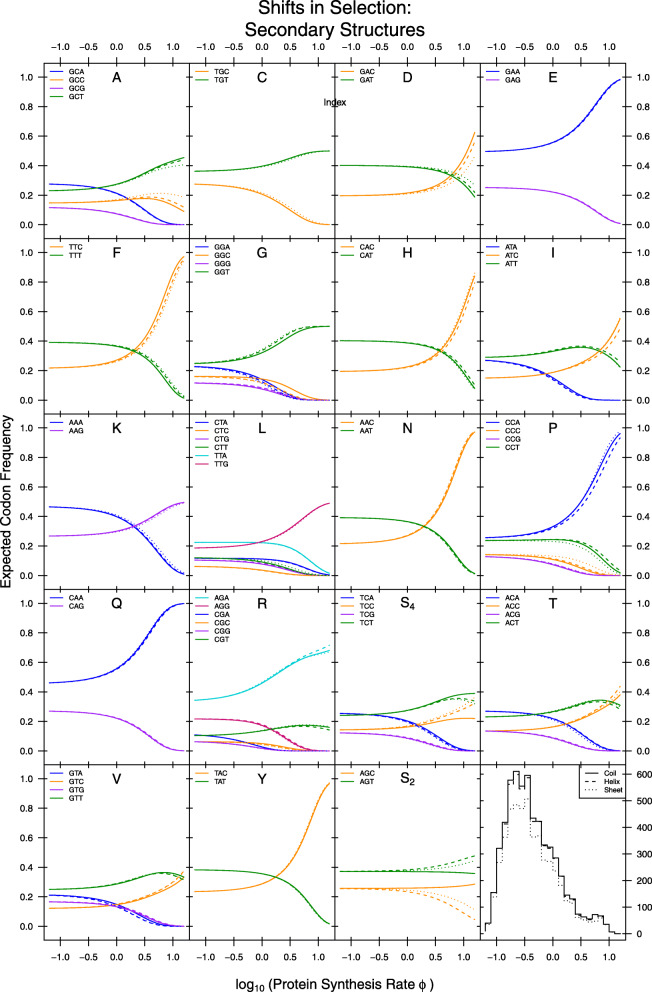
Comparison of expected codon frequencies across protein secondary structures as a function of protein production rates *ϕ*. Expected codon frequencies are estimated using equation 1 (see Materials and Methods). The bottom right histogram gives distributions of protein synthesis rates *ϕ* on the log 10 scale

Although no qualitative (i.e. overall) selective shifts on codon usage were detected, examination of the 95% posterior probability intervals for selection estimates *Δη* indicate clear quantitative differences in the strength of selection related to individual codons. We find that for most of the 18 amino acids with multiple synonyms, selection differs between secondary structures for at least one codon (i.e. its *Δη* 95% posterior probability intervals do not overlap). These differences mostly reflect quantitative changes in the average strength of selection and not a qualitative switch in the most selectively-favored codon. The one qualitative exception to this appears to be serine (S_4_ and S_2_) in coils of *S. cerevisiae*. While codons TCT and AGC are disfavored in helices and/or sheets, this is not the case for coils in which there appears to be no differences in the preference for these two codons and the genome-wide most selectively-favored codons (TCC and AGT, respectively, Fig. 1A,B). However, while we do detect quantitative selective shifts across secondary structures, these shifts are very small and are expected to have little impact on codon frequencies across protein secondary structures (Fig. 2, see Additional File 1, Fig. S4 – S6 for plots of individual structures with observed codon frequencies), especially for genes with average to low expression levels.

### Intrinsically-disordered regions show distinct patterns of selection on codon usage

In *S. cerevisiae*, we found that information on whether a region was structured or intrinsically-disordered better explained intragenic codon usage patterns than protein secondary structures in *S. cerevisiae* (Table 2, Models Y_1_ vs. Y_5_, *Δ*DIC = 220). Consistent with this, selection was 9% weaker, on average, in IDRs compared to structured regions (Deming Regression $\hat {\beta } = 0.905$, 95% CI: 0.823 – 0.988, Fig. S3A). In contrast to *S. cerevisiae*, splitting codons into structured regions and IDRs in *E. coli* did a worse job of explaining intragenic codon usage patterns than secondary structures (Table 2, Model E_5_ vs. E_1_, *Δ*DIC = 343). This was unsurprising given the rarity of IDRs in prokaryotic proteomes [58]. Despite being a worse fit compared to the secondary structure model in *E. coli*, the structured regions vs. IDR model is still a significant improvement over the null model (Tables 1 and 2, Model E_0_ vs. E_5_, *Δ*DIC = 129). Even though the Deming regression slope comparing structured regions and IDRs in *E. coli* was of similar magnitude to the same slope estimated for *S. cerevisiae* (0.905 vs. 0.933, respecitvely), the slope was not significantly different from 1 (Fig. S3B, Deming Regression $\hat {\beta } = 0.933$, 95% CI: 0.849 – 1.020).

**Table 2:** Model comparisons of structure categorizations based on the Deviance Information Criterion (DIC), where the smallest value is considered the best model fit. For simplicity, only models which are an improvement over the model over the best secondary structure model (Table 1) are shown, with the exception of the Structured and IDR model for *E. coli*. H: helix. E: sheet. C: coil. Superscripts *S* and *D* indicate if the secondary structure predictions include predictions from structured regions *S* or IDRs *D*, respectively, i.e. S = H ^*S*^*E*^*S*^*C*^*S*^. R = H ^*D*^*E*^*D*^*C*^*D*^

		Groupings						
Species	Model	I	II	III	IV	V	VI	*Δ*DIC
	Y_10_	H ^*S*^	H ^*D*^	E ^*S*,*D*^	–	C ^*S*^	C ^*D*^	0
	Y_9_	H ^*S*^	H ^*D*^	E ^*S*^	E ^*D*^	C ^*S*^	C ^*D*^	0.46
*S. cerevisiae*	Y_8_	H ^*S*,*D*^	–	E ^*S*,*D*^	–	C ^*S*^	C ^*D*^	83
	Y_7_	H ^*S*,*D*^	–	E ^*S*^	E ^*D*^	C ^*S*^	C ^*D*^	84
	Y_6_	H ^*S*^	–	E ^*S*^	–	C ^*S*^	D	117
	Y_5_	S	–	–	–	–	D	466
	Y_1_	H ^*S*,*D*^	–	E ^*S*,*D*^	–	C ^*S*,*D*^	–	686
	E_6_	H ^*S*^	–	E ^*S*^	–	C ^*S*^	D	0
	E_8_	H ^*S*,*D*^	–	E ^*S*,*D*^	–	C ^*S*^	C ^*D*^	29
*E. coli*	E_10_	H ^*S*^	H ^*D*^	E ^*S*,*D*^	–	C ^*S*^	C ^*D*^	30
	E_7_	H ^*S*,*D*^	–	E ^*S*^	E ^*D*^	C ^*S*^	C ^*D*^	45
	E_9_	H ^*S*^	H ^*D*^	E ^*S*^	E ^*D*^	C ^*S*^	C ^*D*^	47
	E_1_	H ^*S*,*D*^	–	E ^*S*,*D*^	–	C ^*S*,*D*^	–	77
	E_5_	S	–	–	–	–	D	420

Although selection on codon usage was weaker, on average, in IDRs of *S. cerevisiae*, we note a subset of amino acids demonstrate the opposite pattern in which selection against certain codons appears to be stronger: alanine (A), histidine (H), lysine (K), proline (P), and threonine (T) (Fig. S3A). In addition, serine (S_4_ and S_2_) shows shifts in the selectively-favored codon in IDRs relative to structured regions, with the former showing preference for TCT and AGC. This is similar to the results observed in coils for *S. cerevisiae*, but in this case, the selective shifts clearly indicate one of the codons is preferred over the other (i.e. the 95% posterior probability intervals do not overlap with 0, Fig. S3A). Alanine (A), serine (S_4_), and threonine (T) showed a similar pattern in *E. coli* (Fig. S3B). Interestingly, many of these amino acids have a higher propensity for forming disordered regions and/or serve as sites for phosphorylation [59, 60]. Despite the apparent qualitative shift between structured regions IDRs in *S. cerevisiae*, as well as various quantitative shifts, these shifts have very little impact on the expected codon frequencies (Additional File 1, Fig. S7 – S9).

**Fig. 3:**
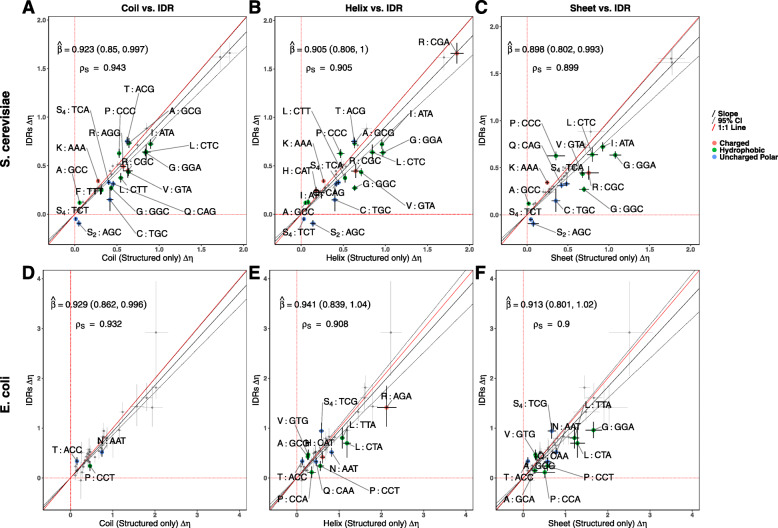
Comparison of selection estimates *Δη* between secondary structures (predicted IDRs removed) and IDRs. Points represent *Δη* values for each codon. Error bars represent the 95% posterior probability intervals. Codons showing significant selective shifts are colored by amino acid property. Negative values indicate a change in the selectively-favored codon relative to the genome-wide most selectively-favored codon. The Deming regression slope *β* and 95% confidence intervals (noted in parentheses) are represented by solid and dashed black lines, respectively. *ρ*_*S*_ indicates the Spearman rank correlation between the *Δη* of the two regions. **(A,D)** Coils vs. IDRs. **(B,E)** Helices vs. IDRs. **(C,F)** Sheets vs. IDRs

We found that categorizing the predicted structured regions (from IUPRED2) based on the corresponding secondary structure predictions (from PsiPred) improved the overall model fits in both species (Models Y_6_ and E_6_), indicating differences in codon usage between secondary structures are not solely due to the presence of IDRs. Comparing selection estimates *Δη* from the secondary structures (with IDRs removed) to *Δη* estimated from IDRs suggests selection on codon usage is, on average, stronger in coil and sheet secondary structures compared to IDRs in *S. cerevisiae* (Additional File 1, Fig. 3). Although a comparison of helices to IDRs has a slope estimate consistent with stronger selection in helices, this slope is not statistically significant (Deming regression $\hat {\beta } = 0.905$, 95% CI: 0.806 – 1.000). In *E. coli*, all three Deming regression slopes are less than 1, but only the comparison between coils and IDRs is statistically significant (Fig. S3F–H). When comparing *Δη* between secondary structures after removing IDRs, we found that many codons still exhibited significant selective shifts between secondary structures (Fig. 3). Notably, the observed selective shifts in *S. cerevisiae* on codons TCT (S_4_) and AGC (S_2_) in coils appears weakened or missing when IDRs are removed, suggesting the previously observed results were driven by differences in selection in IDRs.

Given that all categories of secondary structures were predicted to fall into structured regions or IDRs (Additional File 1, Table S3), we tested whether further dividing up the secondary structures into their corresponding structured and disordered components was better able to explain codon usage variation across regions. Seemingly the most logical split of coils into structured coils (i.e. those likely falling into protein domains) and IDR coils were better model fits than the models that relied solely upon secondary structure or disorder information in both *S. cerevisiae* and *E.coli* (Model Y_8_ and E_8_, respectively). In *S. cerevisiae*, splitting coils into the structured and disordered regions improved upon the model where the IDRs were taken from all three secondary structure classifications in (Model Y_6_ vs. Y_8_, *Δ*DIC = 34), but this model was a worse fit in *E. coli* (Model E_8_ vs. E_6_, *Δ*DIC = 29). Surprisingly, dividing both coils and helices into structured regions and IDRs in *S. cerevisiae* further improved the model fit (Model Y_8_ vs. Y_10_, *Δ*DIC = 83).

### Selection on codon usage varies at the termini of helices in *E. coli*, but not *S. cerevisiae*

Using empirically-determined secondary structures (due to the inaccurate identification of secondary structure boundaries by prediction tools [17, 32]) in *S. cerevisiae*, we found no evidence that natural selection varies at the termini of sheets or coils based on model comparisons via DIC. This was regardless of our choice of the size of the termini (2 or 3 amino acids) or the minimum length of the structure (4 to 7 and 6 to 10 amino acids, respectively; Additional File 1, Table S4 – S5). Regarding the helix secondary structures, only found strong evidence for differences in selection between the core and termini when we used termini of 2 amino acids and included 4 amino acid long structures in our analysis (14 DIC). We note that 4 amino acid structures with 2 amino acid termini don’t actually contain a core section. Further, when we restrict our analyses to secondary structures longer than 4 amino acids, support for differences in selection between termin and core disappeared. For completeness, we note the *Δ*DIC scores were less than 10 DIC units when we restricted our minimum lengths to 5 and 6 amino acid. Excluding 3_10_-helices and *π*-helices had no meaningful impact on these results (Additional File 1, Table S6). Taken altogether, there is evidence selection on codon usage varies between the termini and core of helices in *S. cerevisiae*, but only for very short structures, which seem to be of questionable biological relevance.

Switching our focus to *E. coli*, using empirically-determined secondary structures, DIC-based model comparisons indicate differences at termini relative to the core in helical structures when we restricted the minimum length of helices from 4 to 7 amino acids (Table 3 and Additional File 1, Tables S7 – S7). In this case, we find that *Δη* values for 8 codons are significantly different between the termini and core (*p*=0.25 for one tail binomial test with *n*=120; Additional File 1, Fig. S12). On the other hand, sheets demonstrated variable patterns depending on the length, similar to what we saw with helices in *S. cerevisiae*. When restricting the length to a minimum of 4 or 5 amino acids, DIC indicates there is a difference between the core and termini of sheets (48 and 24 DIC Units, respectively), which corresponds to cores of 0 and 1 amino acid, respectively. As with helices in *S. cerevisiae*, these results with sheets in *E. coli* should be taken with caution given that DIC clearly supports no difference in selection between the core and termini of sheet components with a minimum length 6 and 7 amino acids (37 and 49 DIC Units). The same analyses always favored no differences between the termini and core in coils, though note that the *Δ*DIC scores less than 10 DIC units when we restricted our minimum lengths to ≤ 6 amino acids.

**Table 3:** Comparing models with termini (first and last 2 amino acids, respectively) of secondary structures separated from the core of the structure in *S. cerevisiae* and *E. coli*. Results are for secondary structures of minimum length 6 amino acids. H: helix. E: sheet. C: coil. *Δ*DIC=DIC_*i*_−DIC_Best_. Secondary structures based on empirically-determined secondary structures

			Groupings			
Species	Model	Secondary Structure	I	II	III	*Δ*DIC
	Y _1*a*_		Whole Structure	–	–	0
	Y _1*b*_	H	Termini	Core	–	2
	Y _1*c*_		N-terminus	Core	C-terminus	33
	Y _1*d*_		Whole Structure	–	–	0
*S. cerevisiae*	Y _1*e*_	E	Termini	Core	–	33
	Y _1*f*_		N-terminus	Core	C-terminus	57
	Y _1*g*_		Whole Structure	–	–	0
	Y _1*h*_	C	Termini	Core	–	47
	Y _1*i*_		N-terminus	Core	C-terminus	96
	E _1*b*_		Termini	Core	–	0
	E _1*c*_	H	N-terminus	Core	C-terminus	13
	E _1*a*_		Whole Structure	–	–	64
	E _1*e*_		Whole Structure	–	–	0
*E. coli*	E _1*g*_	E	N-terminus	Core	C-terminus	37
	E _1*f*_		Termini	Core	–	43
	E _1*h*_		Whole Structure	–	–	0
	E _1*i*_	C	Termini	Core	–	6
	E _1*j*_		N-terminus	Core	C-terminus	14

#### Previous claim of selective shifts at the start of helices is due to artifacts

**Fig. 4:**
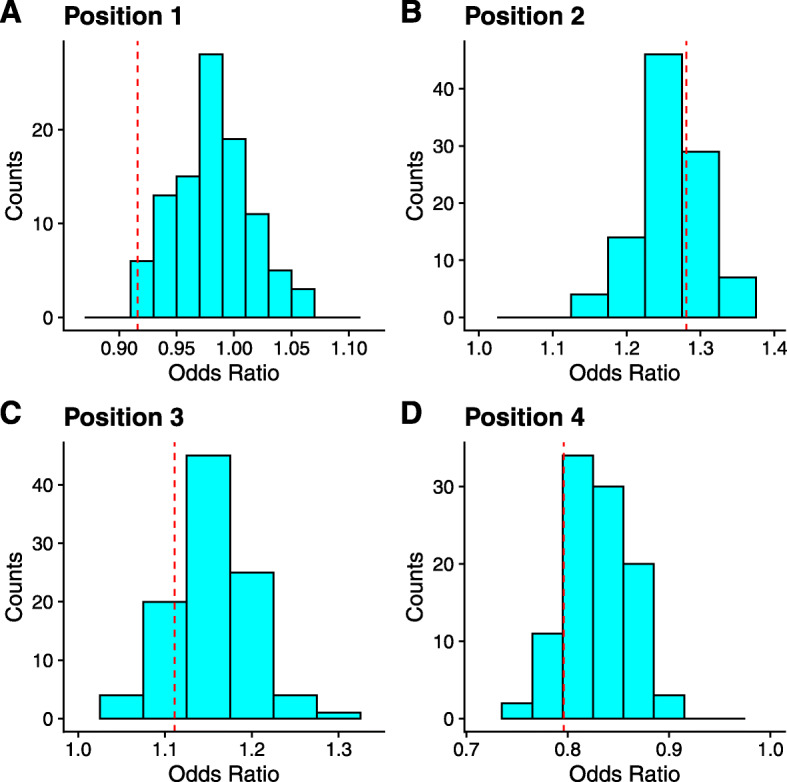
Odds ratio distributions for 100 simulated genomes by relative position in helices. Genomes were simulated assuming selection was identical across all positions in the helices. Dashed red lines indicate the odds ratio reported by [17] estimated from real codon usage patterns. Only proteins used in [17] were included in this analysis

Although we could not entirely rule out that selection on codon usage differed at the termini of helices in *S. cerevisiae* (see above), we found no support for the model allowing for differences in selection on codon usage at the second and third positions of helices relative to the model assuming no differences in selection within helical structures (*Δ*DIC = 30) [17]. Using simulated data that assumes the strength and direction of selection for a codon is constant across the entire genome, we found the odds ratios reported by [17] were within the range of odds ratios generated using the simulated data (Fig. 4). Importantly, these odds ratios are not centered around 1, inconsistent with the expectation under the null commonly used in hypothesis tests with odds ratios. This suggests the enrichment of “optimal” and “non-optimal” codons at positions 1 and 4, and positions 2 and 3, respectively, of helices observed by [17] are an artifact of various confounding factors, such as amino acid biases and gene expression, that can shape codon usage patterns unrelated to natural selection.

## Discussion

The goal of this work is to quantify the general relationship between codon usage and protein structure. This is in contrast to other work which has focused on identifying regions thought to be important to protein structure due to conservation of synonymous codon usage patterns between species [17, 37, 40]. To account for the effects of amino acid biases and gene expression, we used the population genetics-based model ROC-SEMPPR that explicitly includes the effects of mutation bias, selection, and genetic drift on synonymous codon usage patterns. Fitting ROC-SEMPPR to different genic regions allowed us to test for qualitative and quantitative differences in selection on codon usage related to protein structure in *S. cerevisiae* and *E. coli* using both emprically and computationally determined structures. With the exception of serine codons TCT and AGC codons in helices and sheets, we found no evidence for qualitative shifts in the nature of selection across protein secondary structures and IDRs in either species. Instead, we observed a high frequency of small quantitative shifts in the strength of selection for specific codons across different protein structures. Importantly, there were not consistent patterns in the direction of these selective shifts. Our results contrast with previous work [36, 39] which has claimed that certain protein structures show preferences for slow codons: overall, selection on codon usage is highly correlated between protein structures. While our results are consistent with enrichment of a codon within a structure relative to another structure, our results do not indicate differences in the occurrence of systematic reversals in codon preference between regions.

We also find evidence that selection on codon usage varies at the termini of helices in *S. cerevisiae* and *E. coli*. In addition, we also found limited evidence that selection on codon usage varied at the termini of sheets in *E. coli*. However, evidence of differences in selection on codon usage at the termini of secondary structures in both species were sensitive to the minimum lengths (in number of amino acids) of the structures included in these analyses. It is unclear if this indicates a length-dependent effect on selection on codon usage at the termini or a loss of statistical power. We could not exhaustively test all possible minimum length cutoffs or definitions of a secondary structure termini (e.g. the first 2 vs. first 3 amino acids); thus, we cannot exclude the possibility that even some of the stronger evidence of differences in selection on codon usage within secondary structures (e.g. differences between termini and core of helices in *E. coli*) is not due to some artifact. Regardless, while our results are consistent with possible enrichment of certain codons in the termini of certain secondary structures [32], our results do not indicate a systematic changes in codon preference at the termini of these structures.

The codon-specific nature of ROC-SEMPPR’s *Δη* parameter allows the detection of codon-specific difference that may be hidden to other approaches that average over the codon and amino acid usage of a region. For example, our results suggest overall weaker selection on codon usage in IDRs, which is consistent with either relaxation of selection against missense errors in IDRs (as hypothesized by [38]) and/or increased selection for inefficient codons within IDRs to modulate cotranslational folding of upstream structured regions (as hypothezied by [39]). Nevertheless, a subset of amino acids (alanine, glycine, histidine, lysine, proline, serine, and threonine) showed stronger selection between synonymous codons compared to structured regions in both species. All 7 of these amino acids have higher propensities for forming IDRs (e.g. proline, serine, lysine, alanine, glycine) or play common functional roles in IDRs such as serving as sites for phosphorylation (serine and threonine) [59, 60]. We speculate the apparent increased selection against certain codons in IDRs is due to stronger selection against missense errors for these amino acids in IDRs.

Previous work found differences in codon usage between secondary structures were due to the inclusion of IDRs [39]. In contrast, many of the selective shifts between secondary structures we detected remained after removal of IDRs, although the magnitude of these differences was reduced. These conflicting results highlight a potential issue with relying on metrics that average over the codon usage of a region This suggests approaches relying on metrics such as CAI, which is calculated as an average of codon usage across all amino acids within a region, may obfuscate codon-specific selective shifts.

Perhaps the most surprising finding of this work was that splitting up both helices and coils based on disorder predictions provided the best overall model fit in *S. cerevisiae*. Although a “disordered coil” seems like a natural categorization for a protein structure, the phrase “disordered helix” or “disordered sheet” seems contradictory. Because previous work has found that IDRs can form transient secondary structures (particularly helices) under certain conditions [61, 62], this might explain some of the shift we see between “ordered” and “disordered” helicies and sheets. Clearly, our interpretation is highly speculative and further work in this area is needed.

Although we find some evidence of selective shifts at the termini of helical secondary structures in *S. cerevisiae*, we find no evidence supporting selective shifts at positions 2 and 3 of helical secondary structures [17]. The results in [17] is likely due to confounding factors that can also impact codon usage patterns, such as amino acid biases, which can be particularly problematic when using metrics such as tAI [18]. An important feature of our population genetics based approach is revealed from the null distributions generated under the assumption of no selective differences at positions 2 and 3. That is, the true null distribution of odds ratios is not centered around 1 as is usually assumed (Fig. 4). These findings illustrate that while *ad-hoc* approaches to analyzing sequence data can be useful, care must be taken to ensure that the analysis is consistent with the corresponding evolutionary null model [21]. An evolutionary null model is often meant as the expected patterns if a trait were evolving exclusively in the absence of selection (i.e. genetic drift and mutational bias), but in our case, it refers to the patterns expected if the strength and direction of natural selection on codon usage were the same between two regions. As an alternative to purely *ad-hoc* approaches, population genetics approaches can be used for generating evolutionary null distributions for hypothesis testing with *ad-hoc* approaches, as we show here.

Selectively-favored codons identified by ROC-SEMPPR may differ from those identified by other methods that fail to account for the effects of mutational biases and how codon usage changes as a function of gene expression. As previously noted, the selectively-favored codon may not be the most frequently used codon if the strength of mutation bias and genetic drift is strong relative to natural selection, even in the case of highly expressed genes [6, 44, 45]. The normalized translational efficiency (nTE) metric used by [17] to investigate the relationship between codon usage and protein secondary structure is based on the relative supply of tRNA (similar to tAI) and the demand for a tRNA, as estimated by codon usage in the transcriptome based on observed mRNA abundances. The selectively-favored codons for each amino acid identified by ROC-SEMPPR and nTE are in agreement for only 11 of the 19 amino acids (Additional File 1, Fig. S13). A common pattern emerges for the other 8 amino acids: the selectively-favored codon is also mutationally-favored based on ROC-SEMPPR’s parameter estimates. This leads to the odd situation where the supposedly selectively-favored codon according to nTE decreases in frequency as gene expression increases. Even though nTE considers gene expression when estimating demand for a tRNA, it does not consider how codon frequencies change with gene expression or how mutation biases impact codon usage. We suspect this leads to the nTE metric over-penalizing codons that are both mutationally and selectively-favored. We also found that nTE is poorly correlated with empirical ribosome densities, suggesting it is a poor estimate of translation efficiency (Additional File 1, Fig. S14).

Codon usage is predominantly thought to be related to protein structure via modulation of cotranslational folding by altering the speed of translation or by reducing missense errors at sites thought to be important for protein folding [10, 28, 63]. Studies on the association between regions of slow codons and larger protein domains often focus on regions that are 35 or more amino acids downstream from the domain to take the restrictive nature of the ribosome tunnel on domain folding into account (long [37, 40]. In a similar manner, when considering secondary structures, the elongation rate of the codon in the ribosome’s active site will only impact the cotranslational folding of upstream secondary structures. However, it is unclear how large these offsets would need to be given that helical structures can begin forming within the ribosome tunnel [64–66]. To the best of our knowledge, studies on the relationship between codon usage and protein secondary structure, including this one, have not taken into account an offset when examining the relationship between codon usage and seccondary structure. Although this oversight may have little impact if the offset is small, this should be explicitly tested.

Like all models, the biological realism of ROC-SEMPPR is sacrificed for the sake of tractability. The selection coefficients *Δη* estimated using ROC-SEMPPR will reflect the average strength and direction of natural selection on codon usage within a region, including but not limited to selection for translation efficiency, selection for translation accuracy, and selection related to mRNA secondary structure. Given the richness of biological systems, it would be interesting to build upon our analysis and take other factors that might affect CUB into account. For example, some evidence suggests mRNA stem structures occur more frequently in helices and sheets [67]. Faure et al. [68] proposed alterations to elongation rates via mRNA secondary structure could modulate cotranslational protein folding, but previous work has also found that mRNA secondary structure rarely impacts ribosome elongation rates [69]. Regardless, if mRNA secondary structure is correlated with protein secondary structures, then, because we are ignoring it, we expect selection related to mRNA secondary structure to be absorbed into our estimates of *Δη*. In contrast, if mRNA secondary structure is not correlated with protein secondary structure, then selection acting on mRNA secondary structure will contribute to the uncertainty in our estimates of *Δη*. Conceivably, one could add mRNA stability as an additional category when defining different coding regions. A similar analysis could be performed by incoporating knowledge of evolutionarily conserved and variable amino acid sites, with the former hypothesized to be under selection against missense errors at these sites [8, 10]. Such analyses could provide insight into the mechanistic basis of the observed selective shifts; however, these analyses are beyond the scope of our focus.

In addition to mutation bias, another nonadaptive evolutionary force that has been shown to shape codon usage is GC-biased gene conversion generated during meiotic recombination [70]. For the present study, we note that GC-biased gene conversion has previously shown to be present in yeast, but its impact is relatively small [71, 72] and the effects of hitchhiking on codon usage in yeast have been somewhat controversial [73–76]. For other organsisms whose genomes are believe to be more strongly impacted by GC-biased gene conversion, one could take a categorial approach similar to the one we use here and categorize genes by their recombination rate, if known. In theory, ROC-SEMPPR could be expanded to explicitly include GC-biased gene conversion as a quantiative term.

## Conclusions

We find that methods rooted in population genetics can be used to test for shifts in natural selection on codon usage. A key advantage of ROC-SEMPPR is it can be applied to any organism with a sequenced genome, requiring no other input, such as empirical estimates of gene expression [44, 54]. ROC-SEMPPR provides estimates of selection for individual codons, unlike other approaches based on heuristic measures of codon adaptation, such as CAI. We emphasize that we are attempting to quantify the average, genome-wide relationship between selection on codon usage and protein structure. These selective shifts are expected to reflect general mechanisms related to the folding of a protein structure [17, 39]. This is in a similar vein to work that has made broad statements about the preferences of a protein structure for certain codons, such as *α*-helices are preferentially encoded by translationally efficient codons [36]. Our work suggests that such statements are overly-simplistic, as the observed direction and magnitude of selective shifts clearly varies by codon, although these shifts are generally very small. A remaining challenge is to establish the relative importance of the different selective forces that can shape the adaptive evolution of codon usage (e.g. translation efficiency, translation accuracy, mRNA secondary structure) related to protein structure. The direction of natural selection related to these aspects of codon usage do not always operate in the same direction [51, 52]. Future work investigating differences in natural selection on codon usage related to protein folding, protein secretion, and other processes will benefit from the use of such models that are capable of separating out the different selective forces shaping codon usage.

## Methods

Protein-coding sequences (CDS) and amino acids sequences for *S. cerevisiae* S288c (GCF_000146045.2) and *E. coli* K12 MG1655 (GCF_000005845.2) were downloaded from NCBI Refseq. Previous analysis of CUB in *E. coli* indicated approximately 750 genes had outlier codon usage patterns, many of which were hypothesized to be due to horizontal gene transfer [42]. Fitting these outlier genes with ROC-SEMPPR revealed selection on codon usage within these genes was anti-correlated with the remaining genes [48]. Here, our analysis of *E. coli* excludes these outlier genes.

### Identifying protein secondary structure

Our analysis makes use of both protein secondary structures determined empirically via methods like X-ray crystallography, and computationally via methods like PsiPred [57]. The empirical data is a more conservative dataset, with fewer proteins available but more accurate and reliable designations of protein secondary structures. The current implementation of PsiPred has an overall accuracy score of 84% [77], but secondary structure prediction algorithms generally struggle with accurately identifying the termini of secondary structures [17, 32]. Therefore, analyses of codon usage at secondary structure termini were based exclusively on empirically-determined secondary structures.

Empirically-determined protein secondary structures and corresponding protein sequences were obtained from the Protein Data Bank (PDB). Residues were grouped into three overarching structural groups based on their DSSP classification: helix (DSSP H, G, and I), sheet (DSSP E and B), and coil (DSSP S, T, and ’.’). This classification system is consistent with secondary structure prediction algorithms [57] and other analyses of codon usage patterns based on empirically-determined secondary structures [17, 32, 35, 36, 78]. Note that the classification symbol (.) is a catchall containing any amino acids not matching any other DSSP classifications. Protein sequences from PDB were aligned to the *S. cerevisiae* and *E. coli* proteomes using BLAST. Sequences were considered mapped to the proteomes if the PDB sequence covered 80% of the length of the protein and had a percent identity score of 95% or higher. This provided us with 1,097 and 1,285 protein sequences with empirically-determined secondary structures in *S. cerevisiae* and *E. coli*, respectively. This dataset was used for comparing selection on codon usage between and within secondary structures.

Protein secondary structures were predicted for all nuclear protein sequences for *S. cerevisiae* and for 1,742 proteins in *E. coli* using the PsiPred software [57] at default settings. PsiPred combines the secondary structural classifications of DSSP into helices (H), sheets (E), and coils (C).

### Identifying structured and intrinsically-disordered regions

Unlike protein secondary structures, empirically-determined intrinsically-disordered regions (IDRs) are rare. The DisProt database includes only 134 proteins with IDRs for *S. cerevisiae*. Thus, our analysis of codon usage patterns in IDRs and structured regions in *S. cerevisiae* and *E. coli* relied on predicted IDRs using IUPRED2 [79], which provides a quasi-probability of the an amino acid falling into a disordered region, using default settings. An amino acid with a quasi-probability of greater than 0.5 is more likely to be disordered, while a quasi-probability less than 0.5 is more likely to be structured; thus, amino acids with a score less than or equal to 0.5 were classified as structured, while amino acids with a score greater than 0.5 were classified as disordered, consistent with the analysis done by [39].

### Analysis with rOC-SEMPPR

All analyses of CUB was performed using ROC-SEMPPR with the R package AnaCoDa [54]. We note ROC-SEMPPR assumes weak selection. To meet this assumption, serine was split into separate codon groups: the 4 codon group TCN (S_4_) and the 2 codon group AGN (S_2_).

For any amino acid with *n*_*aa*_ synonymous codons, the probability of observing codon *i* in gene *g* can be described by the equation

1 $$\begin{array}{*{20}l} p_{i,g} &= \frac{e^{-\Delta\mathit{M}_{i} - \Delta\eta_{i}\phi_{g}}}{\sum_{j}^{n_{aa}} e^{-\Delta\mathit{M}_{j} - \Delta\eta_{j}\phi_{g}}} \end{array} $$

where *ΔM* represents mutation bias, *Δη* represents natural selection, and *ϕ* represents the evolutionary average protein production rate of gene *g*. Note that *Δ* indicates the mutation bias and natural selection parameters are relative to a reference codon. ROC-SEMPPR’s mutation bias *ΔM* parameter represents the log of the ratio of mutation rates between two synonymous codons [44]. Although originally described as being proportional to relative differences in translation efficiency between two synonymous codons [44], *Δη* can also be interpreted as the critical population genetics parameter *sN*_*e*_, where *N*_*e*_ is the effective population size and *s* represents the selection coefficient relative to the reference codon, here chosen to be the most selectively favored codon for an amino acid. Because ROC-SEMPPR assumes the strength of selection varies with a gene’s expression level *ϕ* and scales this term such that the average level of expression across genes is *ϕ*=1,*Δη* represents the strength and direction of natural selection for a codon in a gene with an average expression level. For genes with lower or higher expression than average, the strength of this selection simply scales with *ϕ*, i.e. *sN*_*e*_=*Δηϕ*.

A deeper understanding of the model parameters can be obtained by considering the cases of no protein production *ϕ*=0 and average protein production *ϕ*=1. We note that ROC-SEMPPR scales *ϕ* such that the *E*[*ϕ*]=1. In the case of no protein production, natural selection on codon usage is completely absent, resulting in mutation biases determining the synonymous codon frequencies. In case of average protein production, the synonymous codon frequencies will reflect the relative strengths and directions of mutation bias and natural selection (proportional to drift). For example, if the mutation bias is stronger and in the opposite direction of natural selection (i.e. mutation and selection favor different codons), then the mutationally-favored codon is expected to be more frequent in an average expression gene. Importantly, *ϕ* scales the strength of natural selection such that strong mutation biases can be (but not necessarily will be) overwhelmed in highly expressed genes. Previous work indicates ROC-SEMPPR’s parameter estimates correlate well with empirical measurements in *S. cerevisiae* and *E. coli* [44, 48].

#### Analysis of selective shifts on codon usage between protein structures

ROC-SEMPPR was fit to all protein-coding sequences in *S. cerevisiae* and *E. coli* to obtain gene-specific estimates of protein production rates *ϕ* and codon-specific estimates of mutation bias *ΔM*. Protein-coding sequences were then partitioned based on the corresponding secondary structure (based on empirically-determined or predicted structures) of each codon/amino acid. This partitioning resulted in FASTA files in which the represented protein-coding sequences contained only one type of protein structure. When fitting ROC-SEMPPR to these data, we estimated the selection coefficients *Δη* while keeping the mutation bias *ΔM* and protein productions rates *ϕ* fixed at their genome-wide estimates, similar to [48]. Our previous work has shown that estimating protein production rates with ROC-SEMPPR instead of using empirical gene expression estimates has little impact on estimates of selection and mutation bias [44]. We also emphasize that empirical gene expression estimates are highly variable across measurements taken from different labs, bringing into question which empirical dataset is best [45]. We previously showed that the distribution of correlation coefficients of gene expression estimates taken from different labs is similar to the distribution of correlation coefficients between ROC-SEMPPR estimated *ϕ* and empirical gene expression estimates (see Supplemental Figure S2 in [48]).

To determine if codon usage patterns are statistically different between protein secondary structures, structural groupings were combined, e.g. helices and sheets (or more specifically, the corresponding FASTA files) were combined into one group (FASTA file) as opposed to treating them as separate groups (FASTA files). These structural groupings were then further merged into different models such that each structure category was represented once, either as a standalone group (e.g. helix) or grouped with another secondary structure (e.g. helix and sheet as one group). To be clear, “different models” simply refers to different ways in which different secondary structures were grouped. This ensured the sequence data (i.e. the number of codons, protein-coding sequences, etc.) is the same across all models, making them directly comparable. We note that the first 35 codons of all genes were excluded to help reduce the impact of a weaker selection on codon usage at the 5’-end of genes [56].

A similar analysis to the one outlined above for comparing codon usage between secondary structures was performed based on the predictions using IUPRED2, in which we compared a model which had structured and disordered regions as separate groupings to a model which treated them as one grouping. Finally, an analysis was performed which combined information from PsiPred and IUPRED2 to classify amino acids based on both methods for classifying structural information. This allowed us to distinguish coils which may be found as part of a larger structured domain from coils part of intrinsically-disordered regions.

#### Analysis of selective shifts on codon usage within protein structures

To examine variation in codon usage within secondary structures, empirically-determined secondary structures were divided into the N-terminus and C-terminus regions, with all codons in between being classified as the core of the secondary structures. To assess robustness of our results, we varied the minimum number of amino acids for a secondary structure as low as 4 in both species, and as high as 10 amino acids in *S. cerevisiae* and as high as 7 amino acids in *E. coli*. We note that the median lengths (in number of amino acids) of helices, sheets, and coils were 10, 4, and 4 (respecitvely) for *S. cerevisiae*, and 9, 4, and 4 (respectively) in *E. coli*. For *S. cerevisiae*, we also varied the termini region to be the first and last 2 or 3 amino acids. To test the hypothesis presented by [17] in *S. cerevisiae*, helices of minimum length 6 amino acids were split up into the second and third codons (relative to the start of the helix) and the remainder of the helix.

#### Comparing model fits and estimates of selection

For statistically comparing codon usage patterns, ROC-SEMPPR model fits were compared using the Deviance Information Criterion (DIC) [80]. Briefly, DIC is a Bayesian information criterion which tries to balance the overall model fit to the data as determined by the posterior distribution and the number of parameters used to fit the data. If the level or nature of selection on codon usage differs between two structures, then it is expected a model treating these structures as separate groupings will have a better (lower) DIC score than model fits treating the structures as single (or merged) groupings. We follow the general rules of thumb for comparing models using information criterion [81]. A model that differs from the minimum DIC model by fewer than 2 DIC units has substantial statistical support. A difference in the range of the 2-4 DIC units are still considered to have strong support, while a difference of 4-7 DIC units are considered to have less support. However, a model that differs from the minimum DIC model by 10 or more DIC units can generally be disregarded. We note that all *Δ*DIC values will represent DIC_*i*_−DIC_Best_, where DIC_*i*_ is the DIC score of the *i*^th^ model and DIC_Best_ is the minimum DIC score (i.e. the best model) of the models under consideration. This means that all reported *Δ*DIC values will fall into the range [0,*∞*).

Comparing models via DIC indicates differences in selection on codon usage between structural regions, but does not tell us how they differ. Similar to [48], we broadly compared codon-specific estimates of selection *Δη* between structural groupings using a model-II regression, which accounts for errors in both the independent and dependent variables [82]. In this work, we used the Deming Regression, as implemented in the R package **deming**. A Deming regression on *Δη* estimated from different structural grouping with a slope of $\hat {\beta } = 1$ (i.e. *y*=*x*) would suggest there is not a general shift in natural selection on codon usage between the two groupings that can be described by the functional relationship *Δη*_*B*_ = *βΔη*_*A*_. On the other hand, a Deming regression slope $\hat {\beta }$ significantly different from 1 is consistent with an overall shift in natural selection on codon usage between the two structural groupings being compared. For each amino acid, its corresponding *Δη* values were scaled relative to the most selectively-favored synonymous codon, i.e. the one most favored by natural selection based on fitting the null model where selection on codon usage does not vary across structural categories; specifically, models *Y*_0_ and *E*_0_ for *S. cerevisiae* and *E. coli*, respectively. As a result, the null model reference codon *Δη* value is always 0 and its synonyms are always *Δη*>0, unless there’s a structure-specific shift in the most selectively-favored codon. In this case, there can be *Δη* values less than 0.

Importantly, the Deming regression only summarizes a possible overall shift in the strength or direction of selection on codon usage between two structural groupings, but does not rule out the possibility that selection is different between specific codons. This information can be obtained by comparing the *Δη* estimates for a codon across the different protein structures. We focus on the codons for which the *Δη* 95% posterior probability intervals do not overlap between structural groupings, as we are most confident in the sign of any shift in selection.

### Simulating codon usage patterns for model validation

To test if we are able to detect shifts in selection across protein regions, we simulated codon usage of two regions under the ROC-SEMPPR model using the empirically determined helices and coils in *S. cerevisiae* (1,097 protein-coding sequences represented) as templates. The codon usage at each amino acid site in the simulated helices, which we refer to as the “Uniform Selection Regions,” is evolving under the same selective pressure. Codon usage in the Uniform Selection Regions was simulated used the *Δη* values, as well as the mutation bias *ΔM* and protein production rates *ϕ*, estimated from a ROC-SEMPPR model fit to the entire set of *S. cerevisiae* protein-coding sequences, excluding mitochondrial sequences. As an example, if we assume these *Δη* values represent selection against translation inefficiency, then the codon usage at all amino acid sites in the Uniform Selection Regions are evolving under selection to reduce translation inefficiency. In contrast, some percentage of amino acid sites (1%, 10%, 50%, 100%) in the simulated coils, which we refer to as the “Heterogeneous Selection Regions,” were randomly selected to be evolving under the opposite selective pressure of the Uniform Selection Regions i.e. the selection coefficients *Δη* at these sites is anticorrelated with the *Δη* used for the Uniform Selection Regions. Building upon our example from above, if the Uniform Selection Regions are evolving entirely under selection to reduce translation inefficiency, then these sites in the Heterogeneous Selection Regions are evolving under selection to increase translation inefficiency. The remaining amino acid sites in the Heterogeneous Selection Regions are evolving under the same selective pressure, i.e. the same selection coefficients *Δη* as the Uniform Selection Regions.

To make sure our approach is robust to factors such as protein structure-specific amino acid biases, we simulated approximately 6,000 *S. cerevisiae* genomes such that the selective pressure was the same across all protein structures (i.e. all structure had the same values of selection parameter *Δη*). Codons from the simulated dataset were assigned to different structures based the computationally predicted structures from PsiPred or IUPRED2. Qualitative and quantitative shifts between helices, sheets, and coils, and between structured regions and IDRs were determined as described in *Comparing model fits and estimates of selection*.

### Evaluating effects of confounding factors in analyses of position-specific codon usage

Previous work found that positions 2 and 3 (relative to the start) of helices in *S. cerevisiae* were enriched in non-optimal codons [17]. To determine if this pattern can be generated by amino acid biases or gene expression, the yeast genome was independently simulated 100 times using AnaCoDa [54] under the ROC-SEMPPR model with the genome-wide selection coefficients *Δη* and mutation bias *ΔM*, as well as the gene-specific estimates of protein production rates *ϕ*, such that the nature of selection was the same for every codon across the genome. Proteins used by [17] for their analysis of position-specific codon usage were pulled and all helices were aligned by position. For each position, enrichment of non-optimal codons based on the nTE metric (as defined in [17]) was tested using a Fisher’s exact test. This generated a distribution of 100 odds ratios per position, which were then compared to the reported odds ratios in [17].

## Supplementary Information

**Additional file 1** PDF also includes supplemental figures and tables referenced in the text.

## Data Availability

Protein-coding sequences for *S. cerevisiae* and *E. coli* are available from National Center for Biotechnology Information (NCBI) RefSeq (https://ftp.ncbi.nlm.nih.gov/genomes/refseq/fungi/Saccharomyces_cerevisiae/latest_assembly_versions/GCF_000146045.2_R64/ and https://ftp.ncbi.nlm.nih.gov/genomes/all/GCF/000/005/845/GCF_000005845.2_ASM584v2/, respectively). All other data and custom scripts are provided via the Github repository (https://github.com/acope3/CUB_Protein_Structure_Analysis). The empirical secondary structures for *S. cerevisiae* and *E. coli* were pulled from a file downloaded from the Protein Data Bank (PDB), but due to a change in the API PDB, this file appears to be no longer available. Due to the size of the original files downloaded from PDB, they are not included in the Github repository (although the relevant files for the organisms are included), but are available upon request.

## Abbreviations

CUB: Codon Usage Bias
ROC-SEMPPR: Ribosome Overcost Stochastic Evolutionary Model of Protein Production Rates
IDR: Intrinsically Disordered Regions
DIC: Deviance Information Criterion
CAI: Codon Adaptation Index
tAI: tRNA Adaptation Index

## References

[1] Hershberg R, Petrov DA (2008). Selection on codon bias. Annu Rev Genet.

[2] Drummond DA, Wilke CO (2009). The evolutionary consequences of erroneous protein synthesis. Nat Rev Genet.

[3] Plotkin JB, Kudla G (2011). Synonymous but not the same: the causes and consequences of codon bias. Nat Rev Genet.

[4] Bulmer M (1991). The selection-mutation-drift theory of synonymous codon usage. Genetics.

[5] Ikemura T (1981). Correlation between the abundance of escherichia coli transfer rnas and the occurrence of the respective codons in its protein genes: A proposal for a synonymous codon choice that is optimal for the e. coli translational system. J Mol Biol.

[6] Shah P, Gilchrist M (2011). Explaining complex codon usage patterns with selection for translational efficiency, mutation bias, and genetic drift. PNAS.

[7] IV TFC, Clark PL. Rare codons cluster. PLoS ONE. 2008; 3. 10.1371/journal.pone.0003412. 10.1371/journal.pone.0003412 PMC2565806 18923675

[8] Akashi H (1994). Synonymous codon usage in drosophila melanogaster: natural selection and translational accuracy. Genetics.

[9] Kurland CG (1992). Translational accuracy and the fitness of bacteria. Annu Rev Genet.

[10] Drummond DA, Wilke CO (2008). Mistranslation-induced protein misfolding as a dominant constraint on coding-sequence evolution. Cell.

[11] Gilchrist MA (2007). Combining models of protein translation and population genetics to predict protein production rates from codon usage patterns. Mol Biol Evol.

[12] Tuller T, Veksler-Lublinsky I, Gazit N, Kupiec M, Ruppin E, Ziv-Ukelson M (2011). Composite effects of gene determinants on the translation speed and density of ribosomes. Genome Biol.

[13] Kudla G, Murray AW, Tollervey D, Plotkin JB (2009). Coding-sequence determinants of expression in escherichia coli. Science.

[14] Hockenberry AJ, Sirer MI, Amaral LAN, Jewett MC (2014). Quantifying position-dependent codon usage bias. Mol Biol Evol.

[15] Peeri M, Tuller T (2020). High-resolution modeling of the selection on local mRNA folding strength in coding sequences across the tree of life. Genome Biol.

[16] O’Brien EP, Ciryam P, Vendruscolo M, Dobson CM (2014). Understanding the influence of codon translation rates on cotranslational protein folding. Acc Chem Res.

[17] Pechmann S, Frydman J (2013). Evolutionary conservation of codon optimality reveals hidden signatures of cotranslational folding. Nat Struct Mol Biol.

[18] Chaney JL, Clark PL (2015). Roles for synonymous codon usage in protein biogenesis. Annu Rev Biophys.

[19] Gould SJ, Lewontin RC (1979). The spandrels of san marco and the panglossian paradigm: A critique of the adaptationist programme. Proc R Soc Lond.

[20] Lynch M. Proceedings of the National Academy of Sciences of the United States of America. 2007; 104:8597–604. 10.1073/pnas.0702207104. 10.1073/pnas.0702207104 PMC1876435 17494740

[21] Koonin EV (2016). Splendor and misery of adaptation, or the importance of neutral null for understanding evolution. BMC Biol.

[22] Geiler-Samerotte KA, Dion MF, Budnik BA, Wang SM, Hartl DL, Drummond DA. Proceedings of the National Academy of Sciences of the United States of America. 2011; 108:680–85. 10.1073/pnas.1017570108. 10.1073/pnas.1017570108 PMC3021021 21187411

[23] Gidalevitz T, Krupinski T, Garcia S, Morimoto RI (2009). Destabilizing protein polymorphisms in the genetic background direct phenotypic expression of mutant sod1 toxicity. PLoS Genet.

[24] Buhr F, Jha S, Thommen M, Mittelstaet J, Kutz F, Schwalbe H, Rodnina MV, Komar AA (2016). Synonymous codons direct cotranslational folding toward different protein conformations. Mol Cell.

[25] Fu J, Murphy KA, Zhou M, Li YH, Lam VH, Tabuloc CA, Chiu JC, Liu Y (2016). Codon usage affects the structure and function of the drosophila circadian clock protein period. Genes Dev.

[26] Holtkamp W, Kokie G, Jager M, Mittelstaet J, Komar AA, Rodnina MV (2015). Cotranslational protein folding on the ribosome monitored in real time. Science.

[27] Walsh IM, Bowman MA, Santarriaga IFS, Rodriguez A, Clark PL. Proceedings of the National Academy of Sciences of the United States of America. 2020; 117:3528–34. 10.1073/pnas.1907126117. 10.1073/pnas.1907126117 PMC7035613 32015130

[28] Yu C, Dang Y, Zhou Z, Wu C, Zhao F, Sachs MS, Liu Y (2015). Codon usage influences the local rate of translation elongation to regulate co-translational protein folding. Mol Cell.

[29] Zhao F, Yu CH, Liu Y (2017). Codon usage regulates protein structure and function by affecting translation elongation speed in drosophila cells. Nucleic Acids Res.

[30] Kimchi-Sarfaty C, Oh JM, Kim IW, Sauna ZE, Calcagno AM, Ambudkar SV, Gottesman MM (2007). A "silent" polymorphism in the mdr1 gene changes substrate specificity. Science.

[31] Simhadri VL, Hamasaki-Katagiri N, Lin BC, Hunt R, Jha S, Tseng SC, Wu A, Bentley AA, Zichel R, Lu Q, Zhu L, Freedberg DI, Monroe DM, Sauna ZE, Peters R, Komar AA, Kimchi-Sarfaty C (2017). Single synonymous mutation in factor ix alters protein properties and underlies haemophilia b. J Med Genet.

[32] Saunders R, Deane CM (2010). Synonymous codon usage influences the local protein structure observed. Nucleic Acids Res.

[33] Brunak S, Engelbrecht J (1996). Protein structure and the sequential structure of mRNA: *α*-helix and *β*-sheet signals at the nucleotide level. Proteins Struct Funct Bioinforma.

[34] Gupta SK, Majumdar S, Bhattacharya TK, Ghosh TC (2000). Studies on the relationships between the synonymous codon usage and protein secondary structural units. Biochem Biophys Res Commun.

[35] Tao X, Dafu D (1998). The relationship between synonymous codon usage and protein structure. FEBS Lett.

[36] Thanaraj TA, Argos P (1996). Protein secondary structural types are differentially coded on messenger rna. Protein Sci.

[37] Chaney JL, Steele A, Carmichael R, Rodriguez A, Specht AT, Ngo K, Li J, Emrich S, Clark PL (2017). Widespread position-specific conservation of synonymous rare codons within coding sequences. PLoS Comput Biol.

[38] Homma K, Noguchi T, Fukuchi S. Codon usage is less optimized in eukaryotic gene segments encoding intrinsically disordered regions than in those encoding structural domains. Nucleic Acids Res. 2016:899. 10.1093/nar/gkw899. 10.1093/nar/gkw899 PMC5137448 27915289

[39] Zhou M, Wang T, Fu J, Xiao G, Liu Y (2015). Nonoptimal codon usage influences protein structure in intrinsically disordered regions. Mol Microbiol.

[40] Jacobs WM, Shakhnovich EI. Proceedings of the National Academy of Sciences of the United States of America. 2017; 114:11434–39. 10.1073/pnas.1705772114. 10.1073/pnas.1705772114 PMC5664504 29073068

[41] Sharp PM, Li W (1987). The codon adaptation index - a measure of directional synonymous codon usage bias, and its potential applications. Nucl Acids Res.

[42] dos Reis M, Wernisch L, Savva R (2003). Unexpected correlations between gene expression and codon usage bias from microarray data for the whole *Escherichia coli* k-12 genome. Nucl Acids Res.

[43] dos Reis M, Savva R, Wernisch L (2004). Solving the riddle of codon usage preferences: a test for translational selection. Nucl Acids Res.

[44] Gilchrist MA, Chen WC, Shah P, Landerer CL, Zaretzki R (2015). Estimating gene expression and codon-specific translational efficiencies, mutation biases, and selection coefficients from genomic data alone. Genome Biol Evol.

[45] Wallace EWJ, Airoldi EM, Drummond DA (2013). Estimating selection on synonymus codon usage from noisy experimental data. Mol Biol Evol.

[46] Xia X (2015). A major controversy in codon-anticodon adaptation resolved by a new codon usage index. Genetics.

[47] Novembre JA (2002). Accounting for background nucleotide composition when measuring codon usage bias. Mol Biol Evol.

[48] Cope AL, Hettich RL, Gilchrist MA. Quantifying codon usage in signal peptides: Gene expression and amino acid usage explain apparent selection for inefficient codons. Biochim Biophys Acta Biomembr. 2018; 1860. 10.1016/j.bbamem.2018.09.010. 10.1016/j.bbamem.2018.09.010 30279149

[49] Zhou T, Weems M, Wilke CO (2009). Translationally optimal codons associate with structurally sensitive sites in proteins. Mol Biol Evol.

[50] Liu H, Rahman SU, Mao Y, Xu X, Tao S (2017). Codon usage bias in 5’ terminal coding sequences reveals distinct enrichment of gene functions. Genomics.

[51] Shah P, Gilchrist MA (2010). Effect of correlated trna abundances on translation errors and evolution of codon usage bias. PLoS Genet.

[52] Stoletzki N (2008). Conflicting selection pressures on synonymous codon use in yeast suggest selection on mRNA secondary structures. BMC Evol Biol.

[53] Landerer C, O’Meara BC, Zaretzki R, Gilchrist MA (2020). Unlocking a signal of introgression from codons in lachancea kluyveri using a mutation-selection model. BMC Evol Biol.

[54] Landerer C, Cope A, Zaretzki R, Gilchrist MA. Anacoda: analyzing codon data with bayesian mixture models. Bioinformatics. 2018:138. 10.1093/bioinformatics/bty138. 10.1093/bioinformatics/bty138 29522124

[55] Gilchrist MA, Wagner A (2006). A model of protein translation including codon bias, nonsense errors, and ribosome recylcing. J Theor Biol.

[56] Gilchrist MA, Shah P, Zaretzki R (2009). Measuring and detecting molecular adaptation in codon usage against nonsense errors during protein translation. Genetics.

[57] Jones DT (1999). Protein secondary structure prediction based on position-specific scoring matrices. J Mol Biol.

[58] Basile W, Salvatore M, Bassot C, Elofsson A. Why do eukaryotic proteins contain more intrinsically disordered regions?PLoS Comput Biol. 2019; 15. 10.1371/journal.pcbi.1007186. 10.1371/journal.pcbi.1007186 PMC6675126 31329574

[59] Singh GP. Association between intrinsic disorder and serine/threonine phosphorylation in mycobacterium tuberculosis. PeerJ. 2015; 2015. 10.7717/peerj.724. 10.7717/peerj.724 PMC4304846 25648268

[60] Uversky VN. The intrinsic disorder alphabet. iii. dual personality of serine. Intrinsically Disordered Proteins. 2015; 3. 10.1080/21690707.2015.1027032. 10.1080/21690707.2015.1027032 PMC5314895 28232888

[61] Bomblies R, Luitz MP, Scanu S, Madl T, Zacharias M (2017). Transient helicity in intrinsically disordered axin-1 studied by nmr spectroscopy and molecular dynamics simulations. PLoS ONE.

[62] Mizuguchi M, Fuju T, Obita T, Ishikawa M, Tsuda M, Tabuchi A (2014). Transient *α*-helices in the disordered rpel motifs of the serum response factor coactivator mkl1. Sci Rep.

[63] Mordret E, Dahan O, Asraf O, Rak R, Yehonadav A, Barnabas GD, Cox J, Geiger T, Lindner AB, Pilpel Y (2019). Systematic detection of amino acid substitutions in proteomes reveals mechanistic basis of ribosome errors and selection for translation fidelity. Mol Cell.

[64] Kramer G, Boehringer D, Ban N, Bukau B (2009). The ribosome as a platform for co-translational processing, folding and targeting of newly synthesized proteins. Nat Struct Mol Biol.

[65] Waudby CA, Dobson CM, Christodoulou J (2019). Nature and regulation of protein folding on the ribosome. Trends Biochem Sci.

[66] Agirrezabala X, Samatova E, Macher M, Liutkute M, Maiti M, Gil-Carton D, Novacek J, Valle M, Rodnina MV. A switch from *α*-helical to *β*-strand conformation during co-translational protein folding. EMBO J. 2022:109175. 10.15252/EMBJ.2021109175. 10.15252/embj.2021109175 PMC8844987 34994471

[67] Jia M, Luo L, Liu C (2004). Statistical correlation between protein secondary structure and messenger rna stem-loop structure. Biopolymers.

[68] Faure G, Ogurtsov AY, Shabalina SA, Koonin EV (2016). Role of mRNA structure in the control of protein folding. Nucleic Acids Res.

[69] Campo CD, Bartholomäus A, Fedyunin I, Ignatova Z (2015). Secondary structure across the bacterial transcriptome reveals versatile roles in mRNA regulation and function. PLoS Genet.

[70] Duret L, Galtier N (2009). Biased gene conversion and the evolution of mammalian genomic landscapes. Annu Rev Genomics Hum Genet.

[71] Harrison RJ, Charlesworth B (2011). Biased gene conversion affects patterns of codon usage and amino acid usage in the saccharomyces sensu stricto group of yeasts. Mol Biol Evol.

[72] Lesecque Y, Mouchiroud D, Duret L (2013). Gc-biased gene conversion in yeast is specifically associated with crossovers: Molecular mechanisms and evolutionary significance. Mol Biol Evol.

[73] Kliman RM, Irving N, Santiago M (2003). Selection conflicts, gene expression, and codon usage trends in yeast. J Mol Evol.

[74] Qin H, Wu WB, Kreitman JMCM, Li W (2004). Intragenic spatial patterns of codon usage bias in prokaryotic and eukaryotic genomes. Genetics.

[75] Pál C, Papp B, Hurst LD (2001). Does the recombination rate affect the efficiency of purifying selection? the yeast genome provides a partial answer. Mol Biol Evol.

[76] Zhou T, Lu ZH, Sun X. The correlation between recombination rate and codon bias in yeast mainly results from mutational bias associated with recombination rather than hill-robertson interference. In: Annual International Conference of the IEEE Engineering in Medicine and Biology - Proceedings 7 VOLS: 2005. p. 4787–90. 10.1109/iembs.2005.1615542. 10.1109/IEMBS.2005.1615542 17281312

[77] Buchan DWA, Jones DT. The psipred protein analysis workbench: 20 years on. Nucleic Acids Res. 2019; 47. 10.1093/nar/gkz297. 10.1093/nar/gkz297 PMC6602445 31251384

[78] Adzhubei AA, Adzhubeib IA, Krasheninnikov IA, Neidle S (1996). Non-random usage of ‘degenerate’ codons is related to protein three-dimensional structure. FEBS Lett.

[79] Mészáros B, Erdős G, Dosztányi Z (2018). Iupred2a: context-dependent prediction of protein disorder as a function of redox state and protein binding. Nucleic Acids Res.

[80] Spiegelhalter DJ, Best NG, Carlin BP, Linde AVD (2002). Bayesian measures of model complexity and fit. J R Stat Soc Ser B Stat Methodol.

[81] Burnham KP, Anderson DR (2004). Multimodel inference: Understanding aic and bic in model selection. Sociol Methods Res.

[82] Sokal RR, Rohlf FJ (1995). Biometry - The Principles and Practices of Statistics in Biological Research.

