# Supplementary material for: Quantifying shifts in natural selection on codon usage between protein regions: a population genetics approach
Source: BMC Genomics. 2022 May 30;23:408. doi: 10.1186/s12864-022-08635-0 (PMC9153123; doi:10.1186/s12864-022-08635-0)
Supplement: Supplementary file 1 — Additional file 1 PDF also includes supplemental figures and tables referenced in the text. [file 12864_2022_8635_MOESM1_ESM.pdf]

Supplemental: Quantifying shifts in natural selection on codon usage between protein regions: A population genetics approach

Alexander L. Cope[1,+] and Michael A. Gilchrist[1,2,3,*]

[1]Genome Science and Technology, University of Tennessee, Knoxville

[2]Department of Ecology and Evolutionary Biology, University of Tennessee, Knoxville

[3]National Institute for Mathematical and Biological Synthesis, Knoxville, TN

[*]Corresponding Author: mikeg@utk.edu

[+]Current: Department of Genetics, Rutgers University

Friday 21st January, 2022

Supplemental Figures

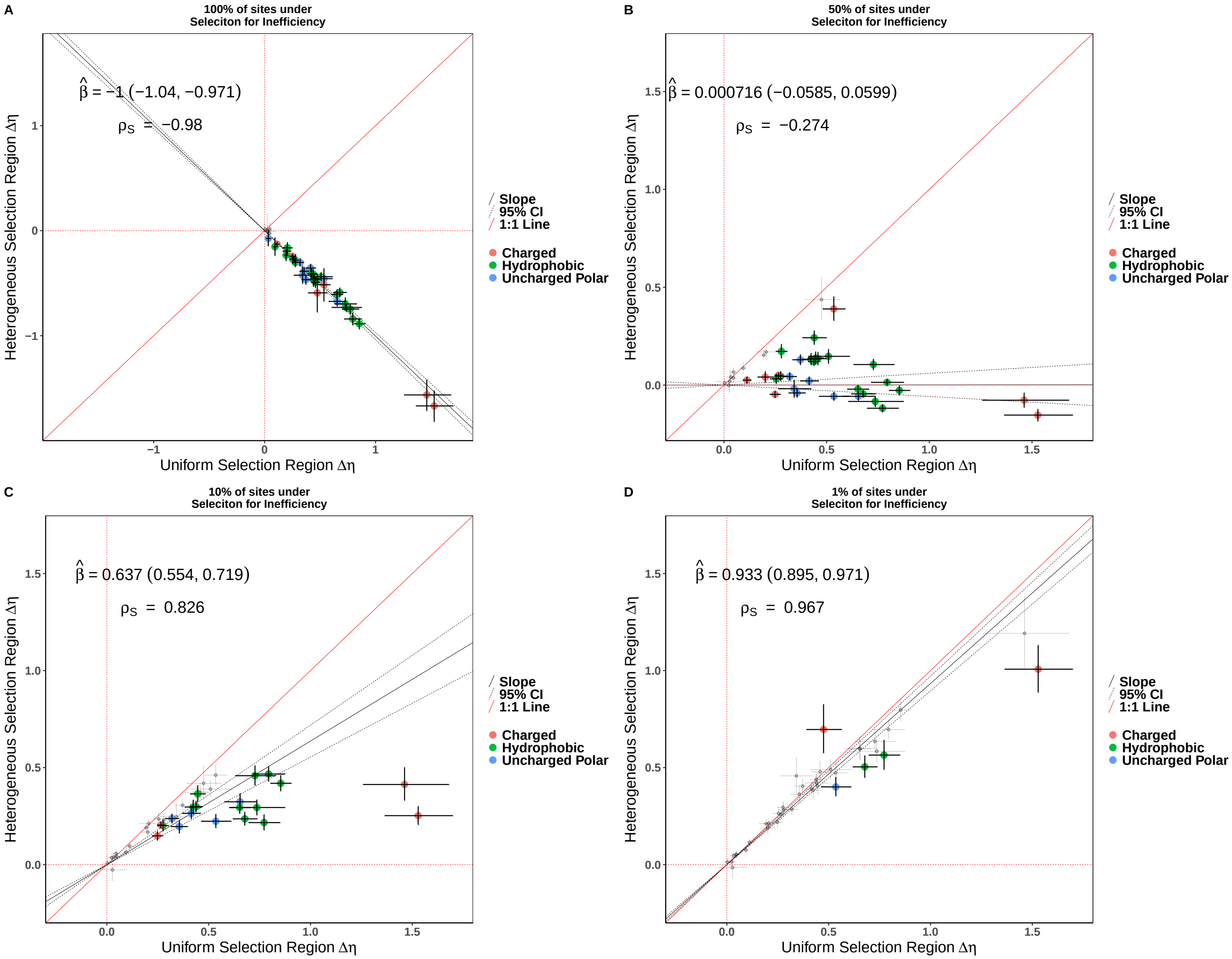

Figure S1: Comparing selection coefficients $\Delta\eta$ between two regions with simulated codon usage. Empirically-determined helices and coils in *S. cerevisiae* (1,097 genes) were used as templates for simulating codon usage. Codon usage at all amino acid sites in the Uniform Selection Regions is evolving under selection against translation inefficiency. Codon usage at a varying percentage of amino acid sites in the Heterogeneous Selection Regions evolving under selection for translation inefficiency. Note that this means these codons have $\Delta\eta$ values perfectly anti-correlated with the $\Delta\eta$ for the rest of the simulated genes. When 50% of the Heterogeneous Selection Regions is under selection for translation inefficiency with the remaining codons under selection against inefficiency, ROC-SEMPPR struggles to identify the most selectively-favord codon. Percentage of sites in Heterogeneous Selection Region under selection for inefficiency. (A) 100%. (B) 50%. (C) 10%. (D) 1%.

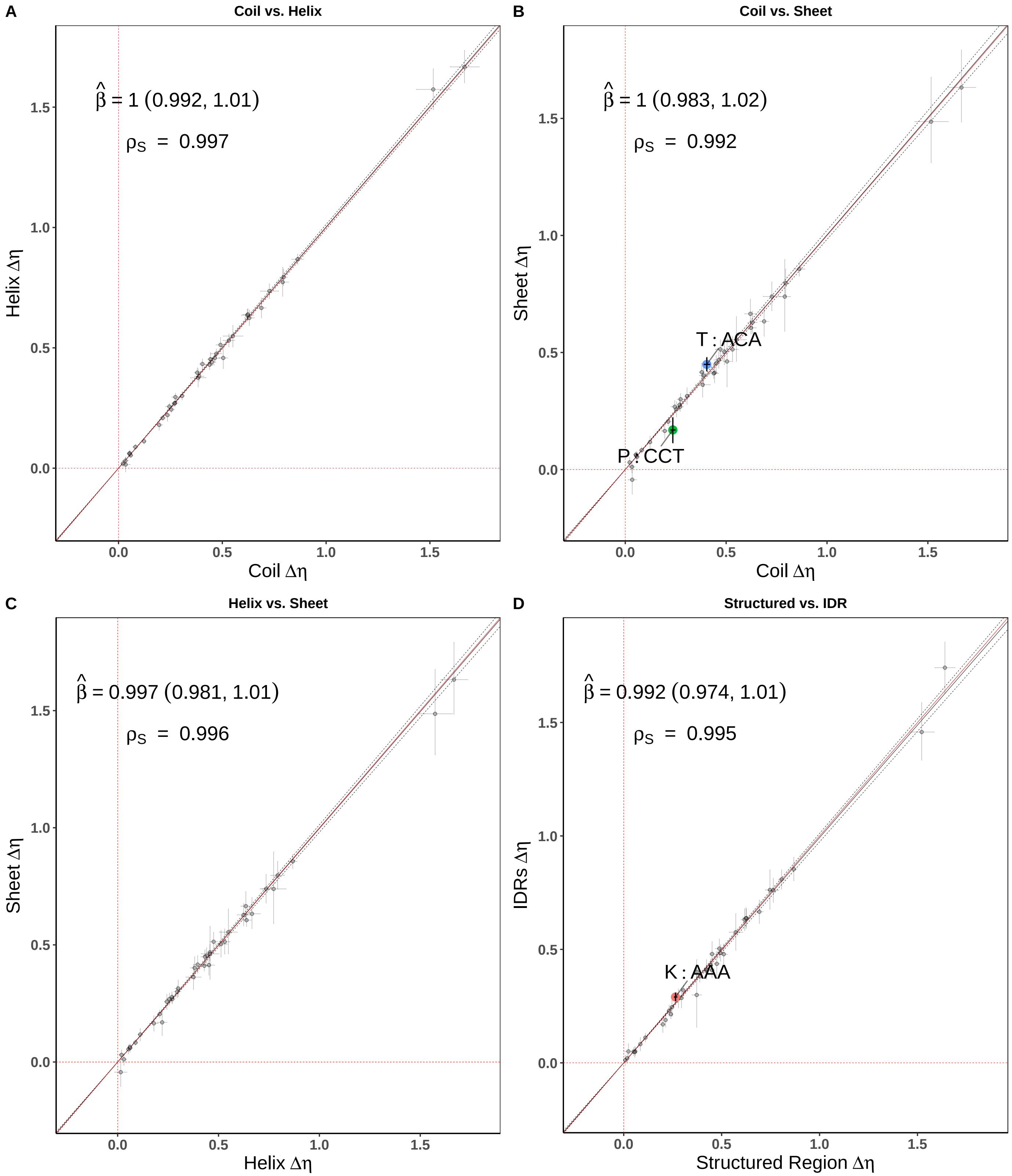

Figure S2: Comparison of selection coefficients $\Delta\eta$ estimated from simulated codon usage for different structures using *S. cerevisiae* protein-coding sequences. All sequences were simulated assuming $\Delta\eta$ was the same across protein structures. The Deming regression slope β and 95% confidence intervals (noted in parentheses) are represented by solid and dashed black lines, respectively. ρ_S indicates the Spearman rank correlation between the $\Delta\eta$ of the two regions. **(A)** Coil vs. Helix. **(B)** Coil vs. Sheet. **(C)** Helix vs. Sheet. **(D)** Structured vs. IDRs

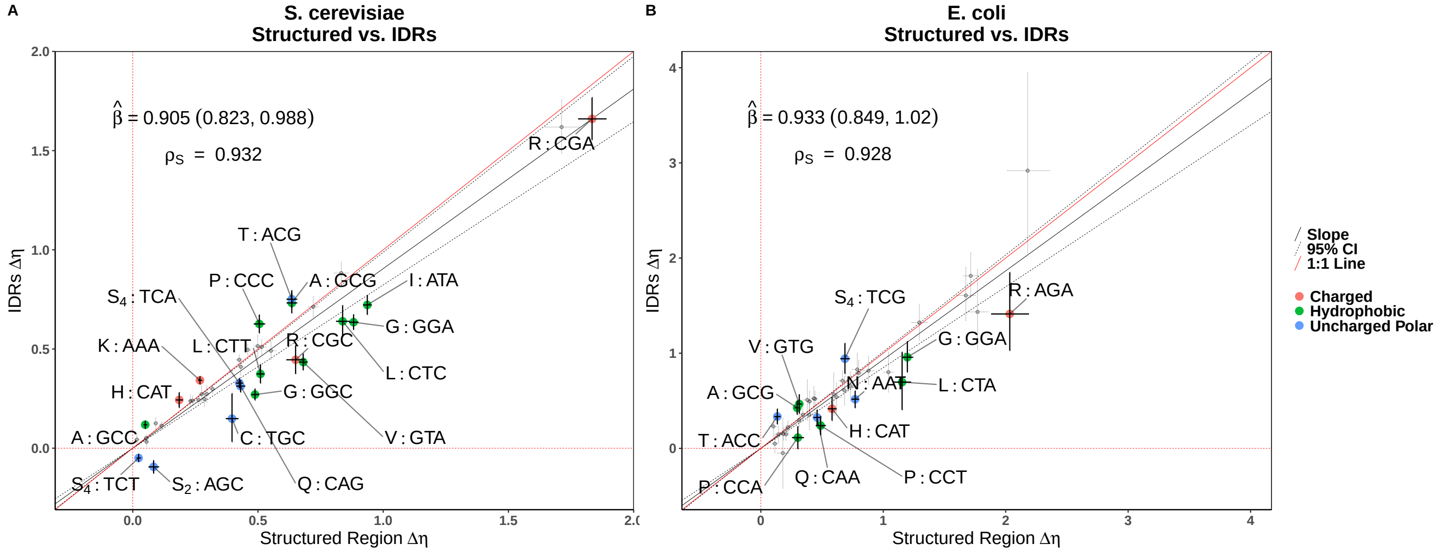

Figure S3: Comparison of selection estimates $\Delta\eta$ between structured regions and IDRs. Points represent $\Delta\eta$ values for each codon. Error bars represent the 95% posterior probability intervals. Codons showing significant selective shifts are colored by amino acid property. Negative values indicate a change in the selectively-favored codon relative to the genome-wide most-selectively favored codon. The Deming regression slope β and 95% confidence intervals (noted in parentheses) are represented by solid and dashed black lines, respectively. ρ_S indicates the Spearman rank correlation between the $\Delta\eta$ of the two regions. **(A,B)** Structured vs. IDRs.

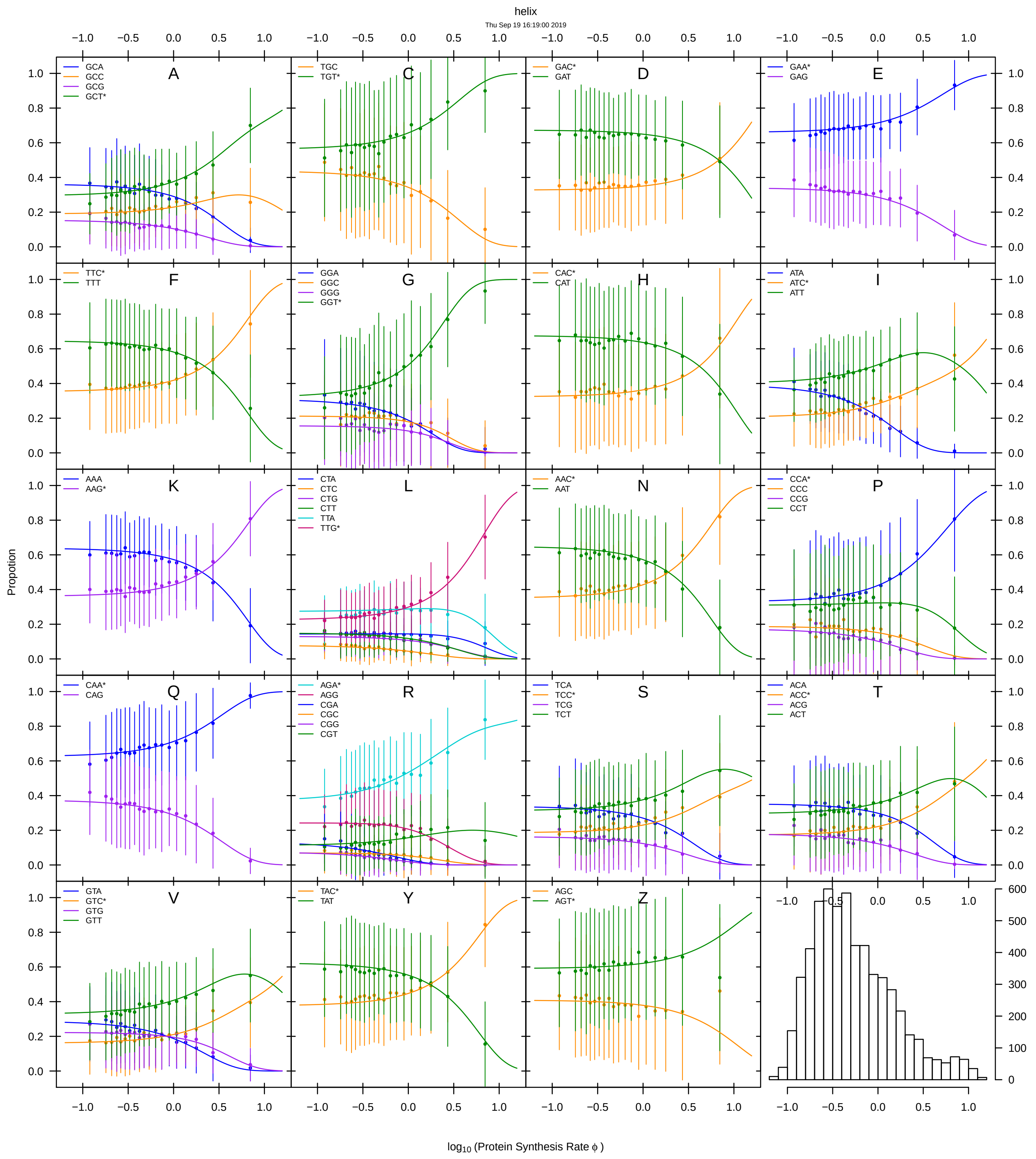

Figure S4: Codon frequencies in predicted helices as a function of protein production rates ϕ. Points and error bars represent the mean and ± 1 standard deviation, respectively, of codon frequencies in protein-coding sequences binned by ϕ. Expected codon frequencies are estimated using equation 1 (see Materials and Methods). Note that $\mathrm{S} = \mathrm{S}_4$ and $\mathrm{Z} = \mathrm{S}_2$.

.

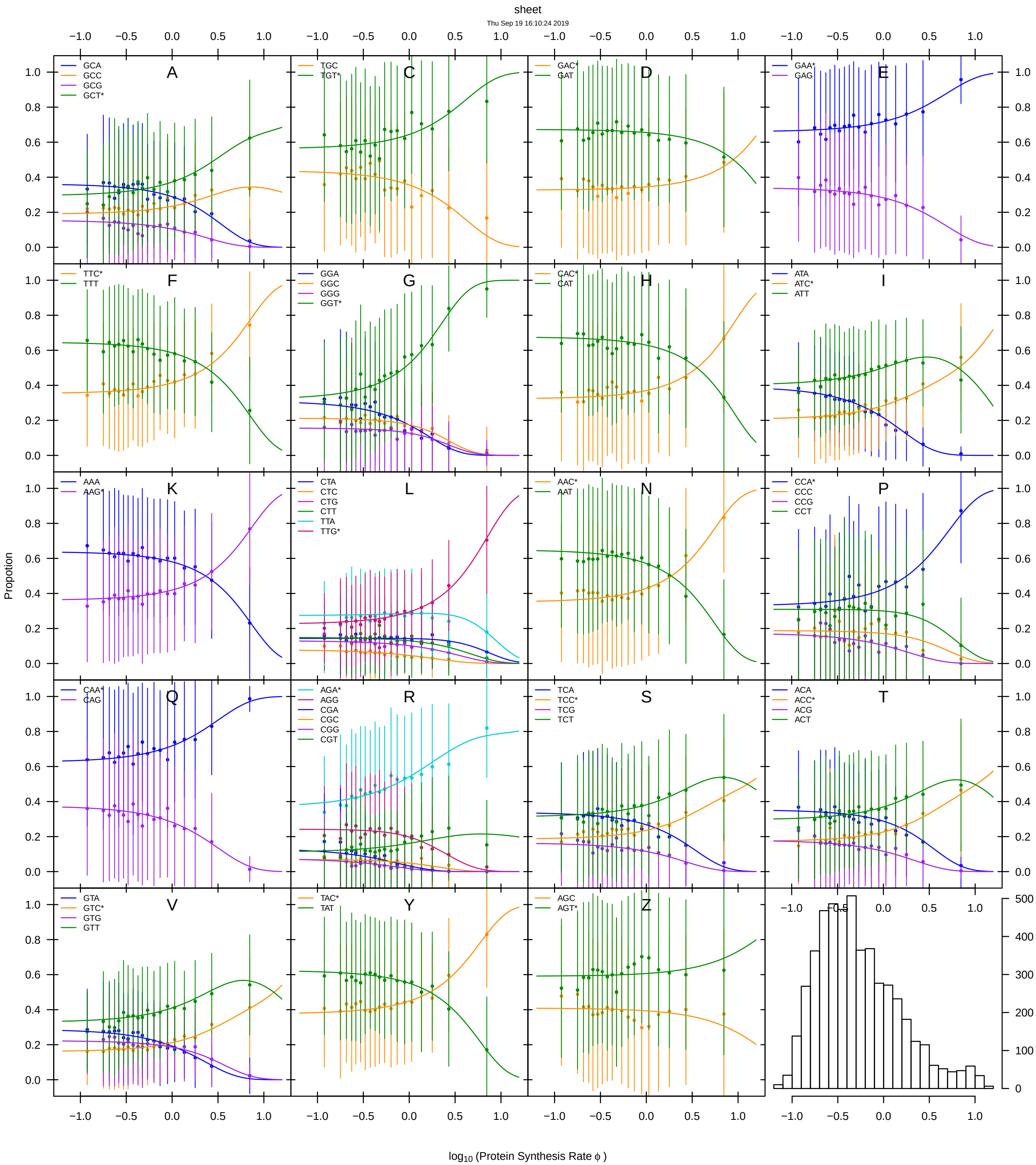

Figure S5: Codon frequencies in predicted sheets as a function of protein production rates ϕ. Points and error bars represent the mean and ± 1 standard deviation, respectively, of codon frequencies in protein-coding sequences binned by ϕ. Expected codon frequencies are estimated using equation 1 (see Materials and Methods). Note that $\mathrm{S} = \mathrm{S}_4$ and $\mathrm{Z} = \mathrm{S}_2$.

.

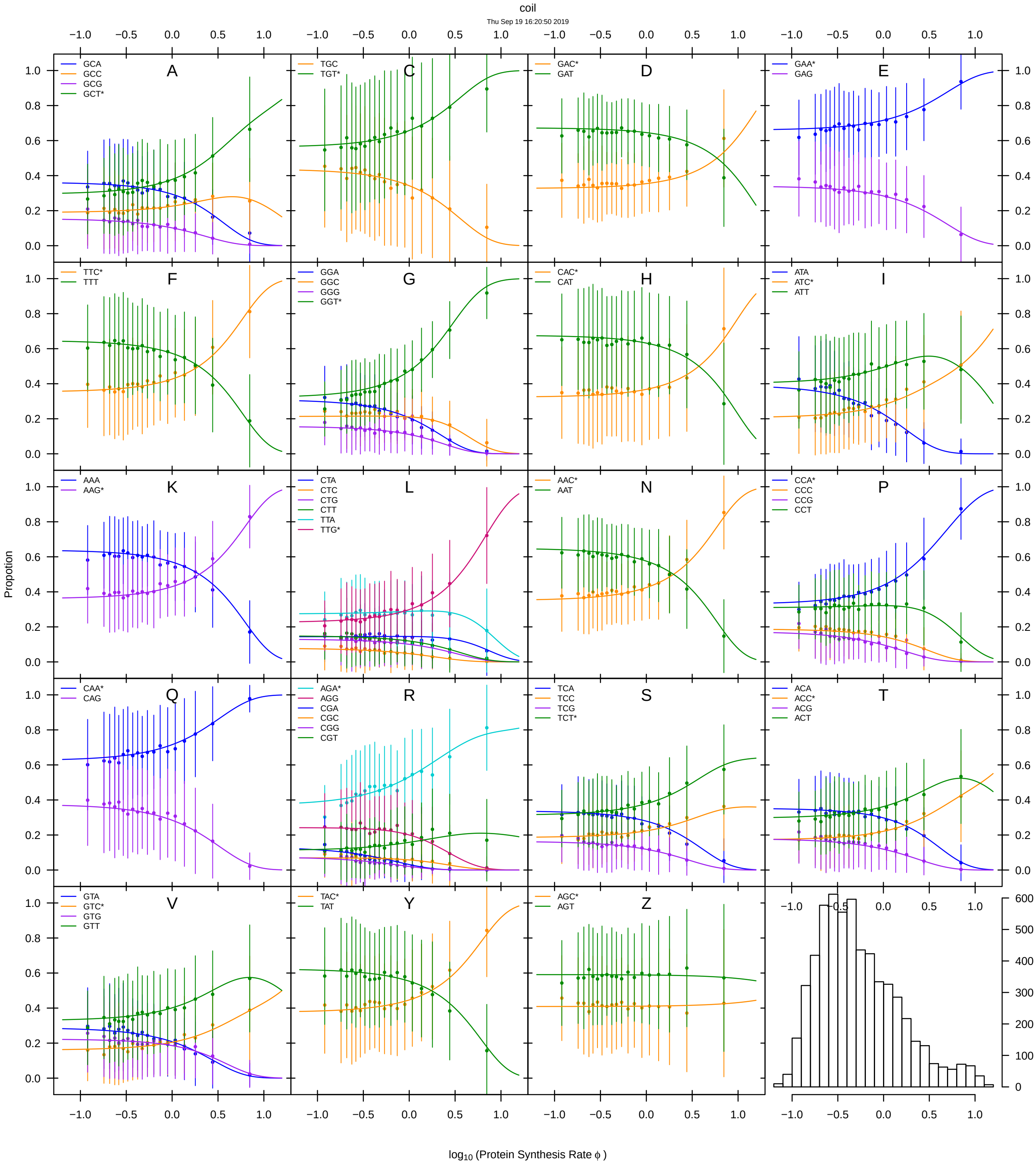

Figure S6: Codon frequencies in predicted coils as a function of protein production rates ϕ. Points and error bars represent the mean and ± 1 standard deviation, respectively, of codon frequencies in protein-coding sequences binned by ϕ. Expected codon frequencies are estimated using equation 1 (see Materials and Methods). Note that $\mathrm{S} = \mathrm{S}_4$ and $\mathrm{Z} = \mathrm{S}_2$.

.

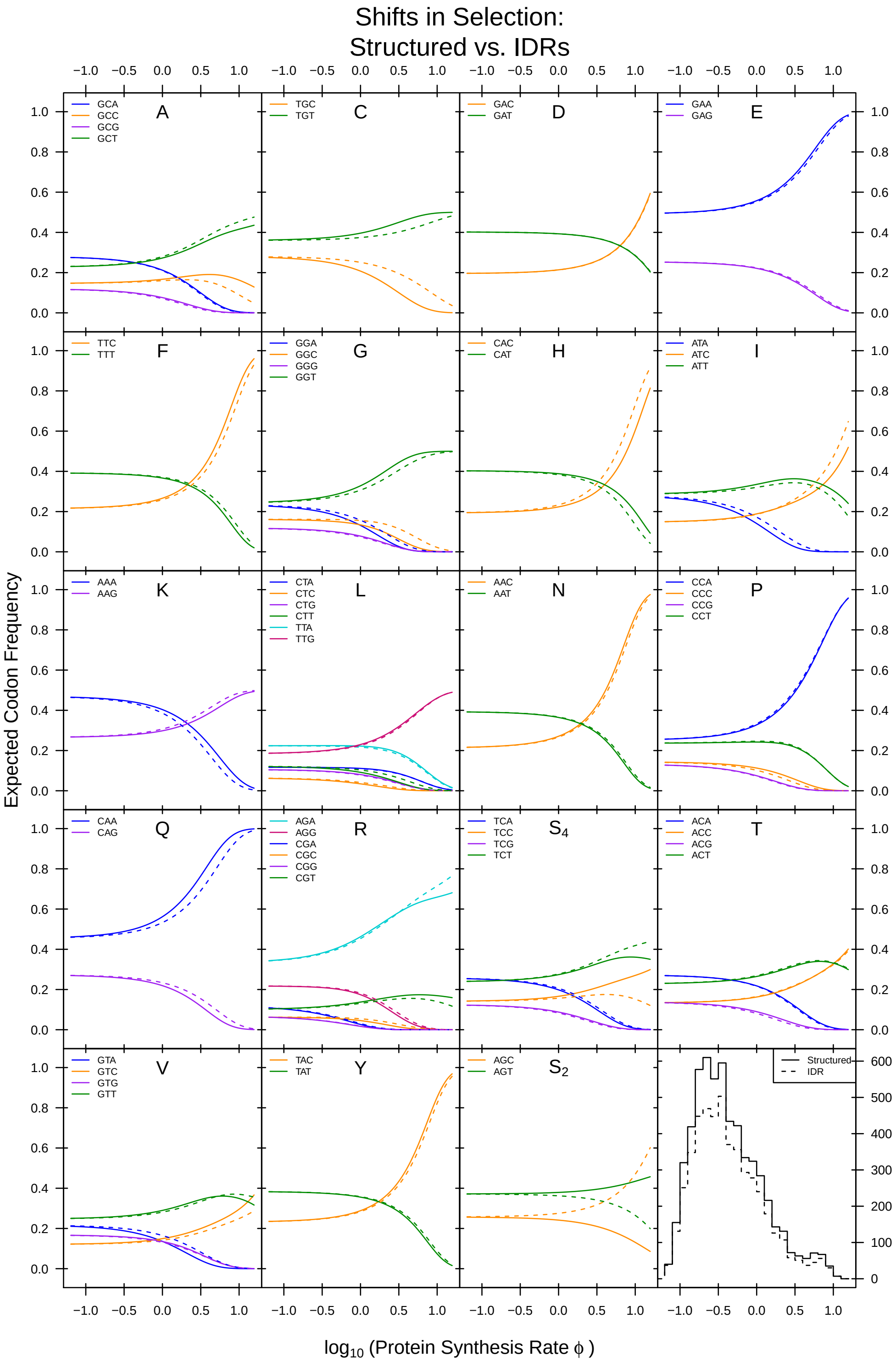

Figure S7: Comparison of expected codon frequencies in structured regions and IDRs as a function of protein production rates ϕ. Expected codon frequencies are estimated using equation 1 (see Materials and Methods).

.

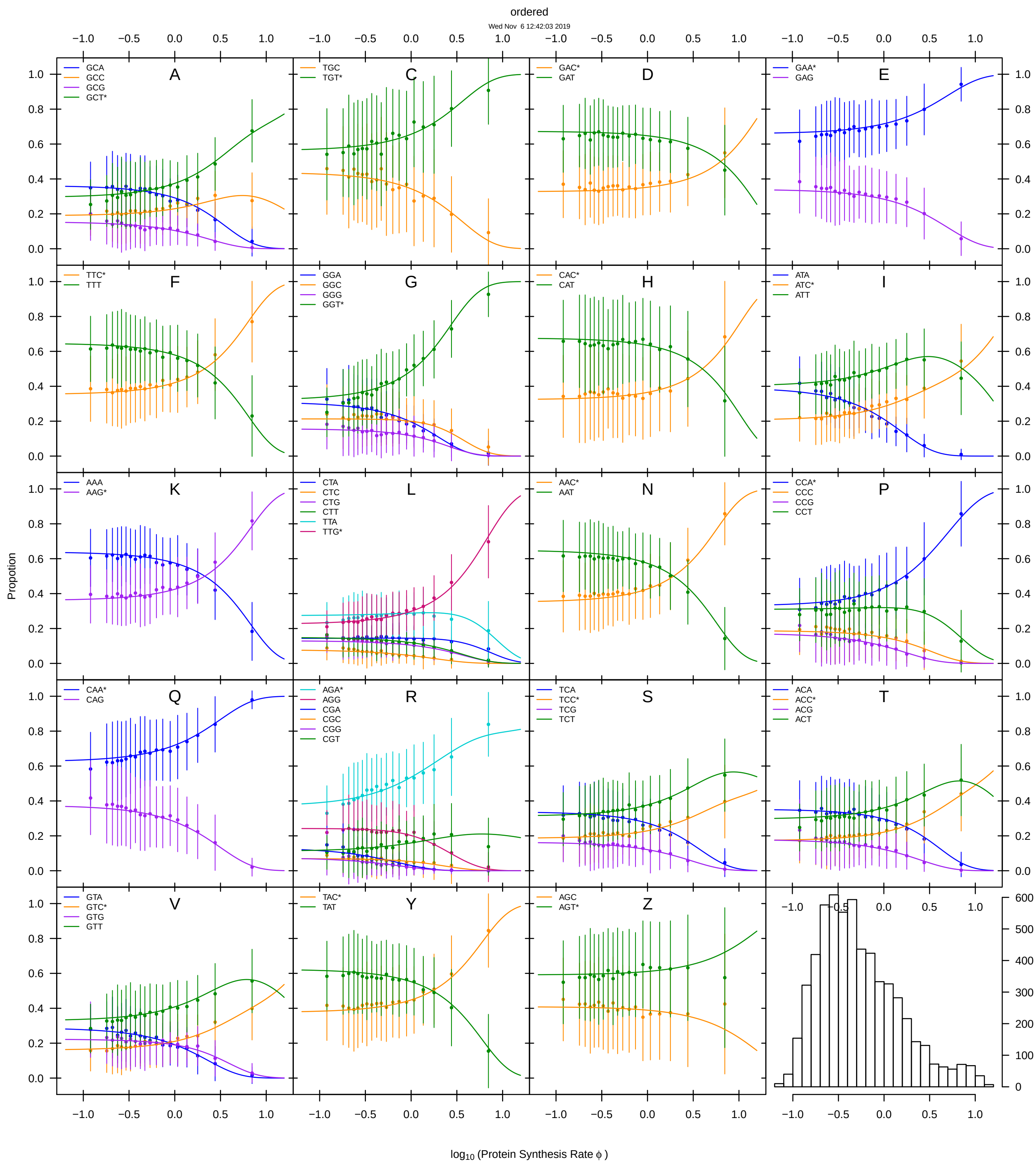

Figure S8: Codon frequencies in predicted structured regions as a function of protein production rates ϕ. Points and error bars represent the mean and ± 1 standard deviation, respectively, of codon frequencies in protein-coding sequences binned by ϕ. Expected codon frequencies are estimated using equation 1 (see Materials and Methods). Note that S = S_4 and Z = S_2.

.

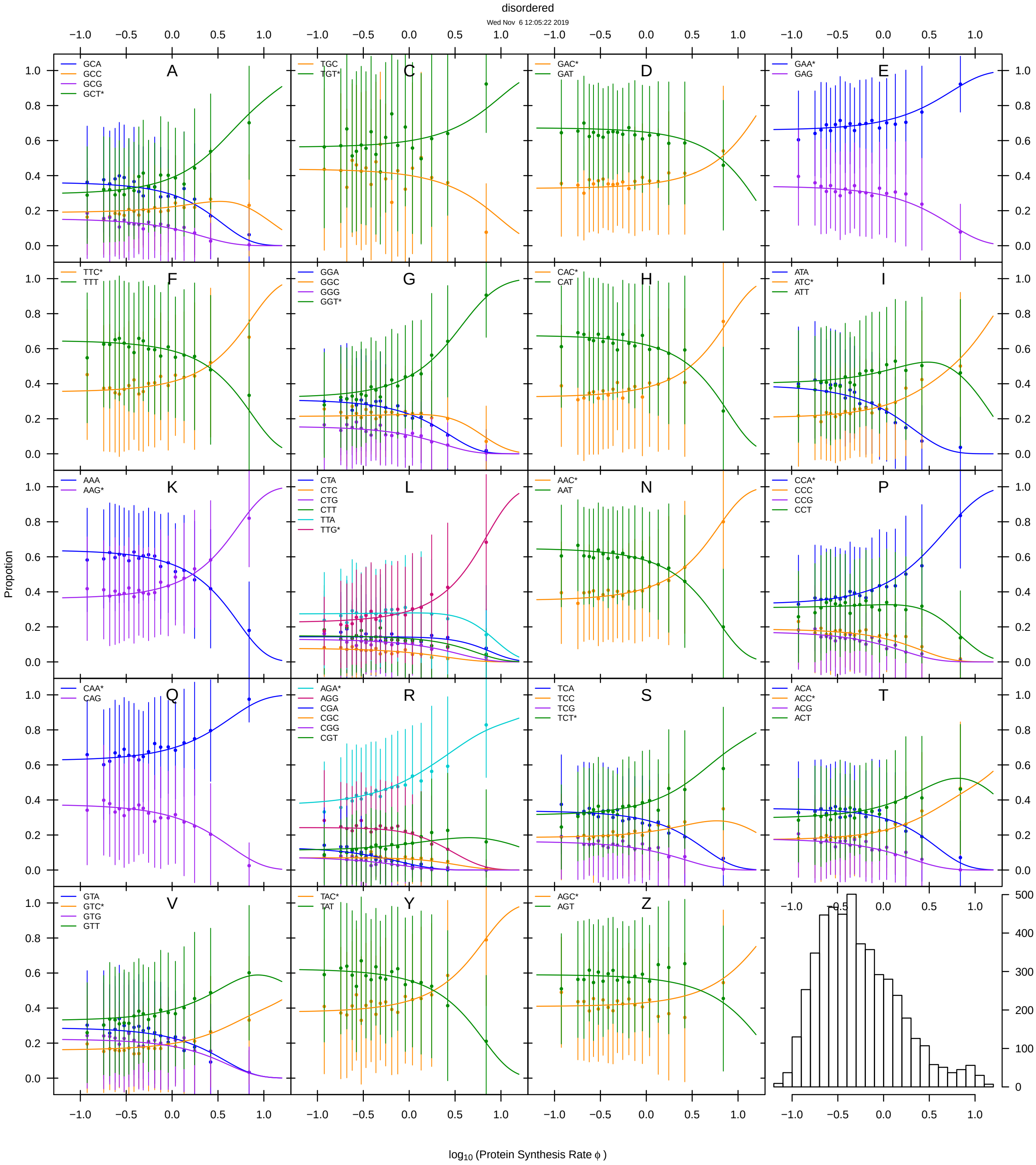

Figure S9: Codon frequencies in predicted intrinsically-disordered regions as a function of protein production rates ϕ. Points and error bars represent the mean and ± 1 standard deviation, respectively, of codon frequencies in protein-coding sequences binned by ϕ. Expected codon frequencies are estimated using equation 1 (see Materials and Methods). Note that S = S_4 and Z = S_2.

.

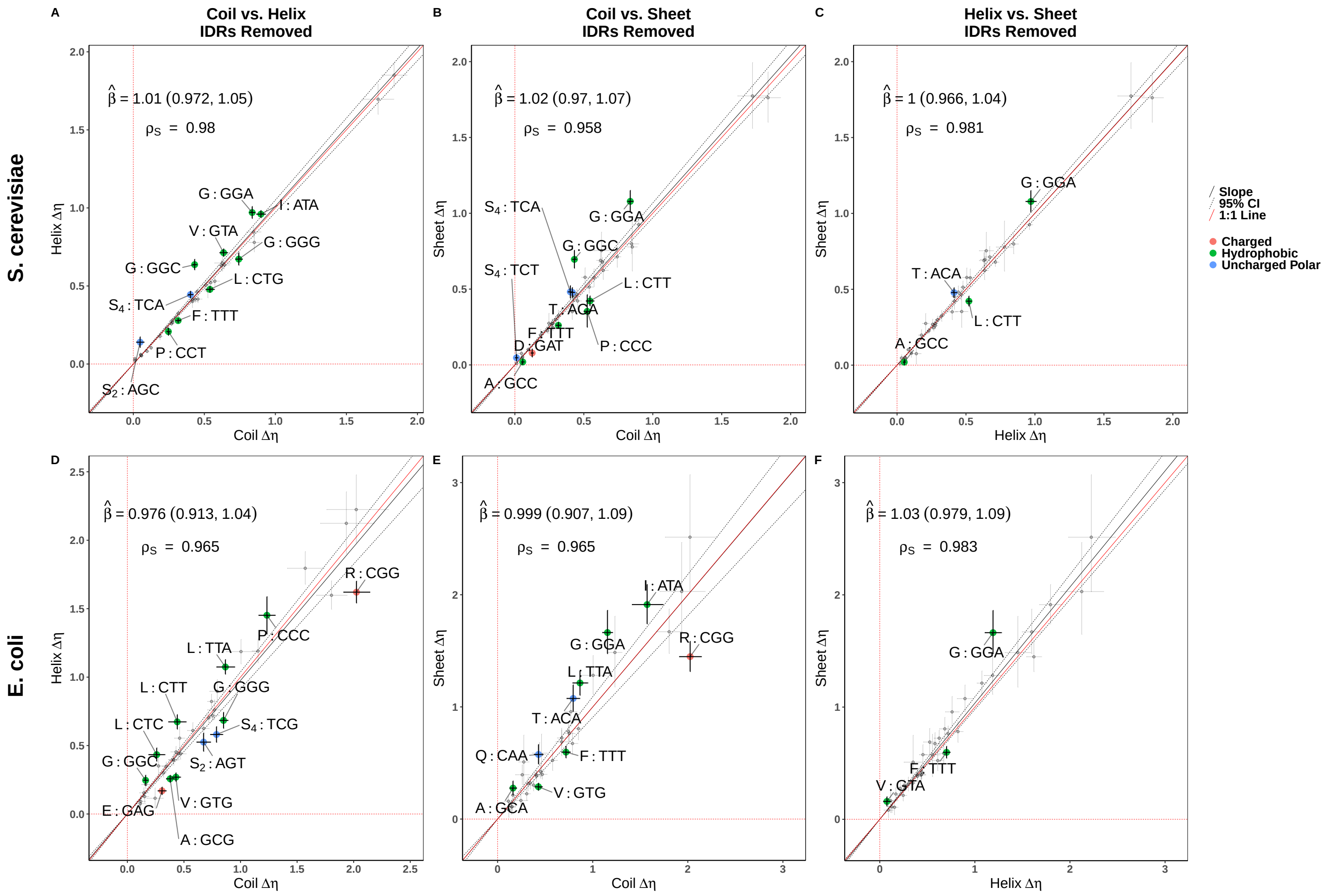

Figure S10: Comparison of selection estimates $\Delta\eta$ between different protein secondary structures with IDRs removed. $\Delta\eta$ is rescaled relative to the genome-wide most selectively-favored codon. Points represent $\Delta\eta$ values for each codon, while error bars represent the 95% posterior probability intervals. Codons showing significant shifts in selection are colored by amino acid property. Negative values indicate a change in the selectively-favored codon relative to the genome-wide most selectively-favored codon. The Deming regression slope and 95% confidence intervals are represented by solid and dashed black lines, respectively. **(A,D)** Coils vs. Helices. **(B,E)** Coils vs. Sheets. **(D,F)** Helices vs. Sheets.

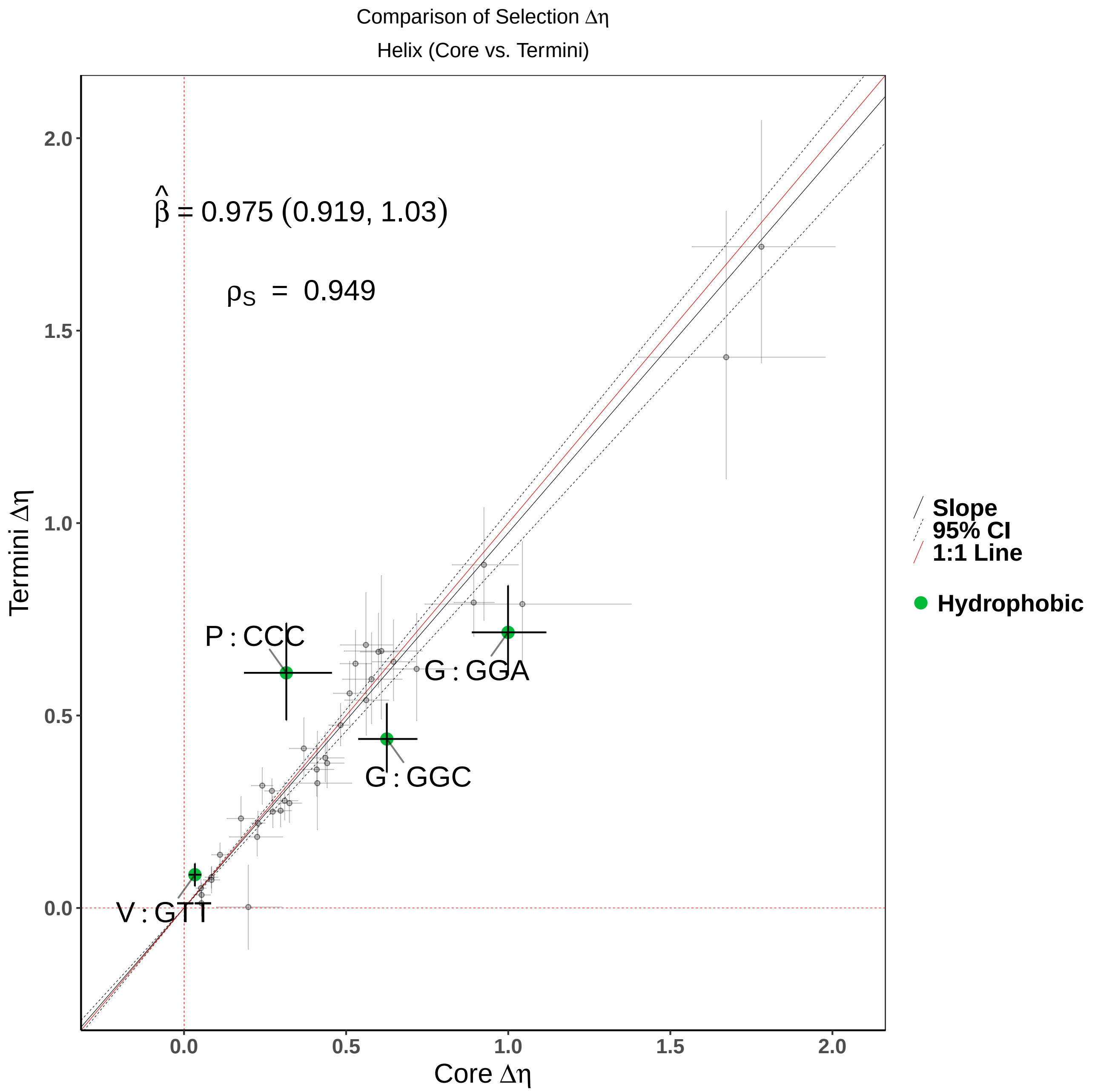

Figure S11: Comparison of selection $\Delta\eta$ between the termini and the core of helices. Data was restricted to helices of minimum length 4 and treating the termini as the first and last 2 amino acids.

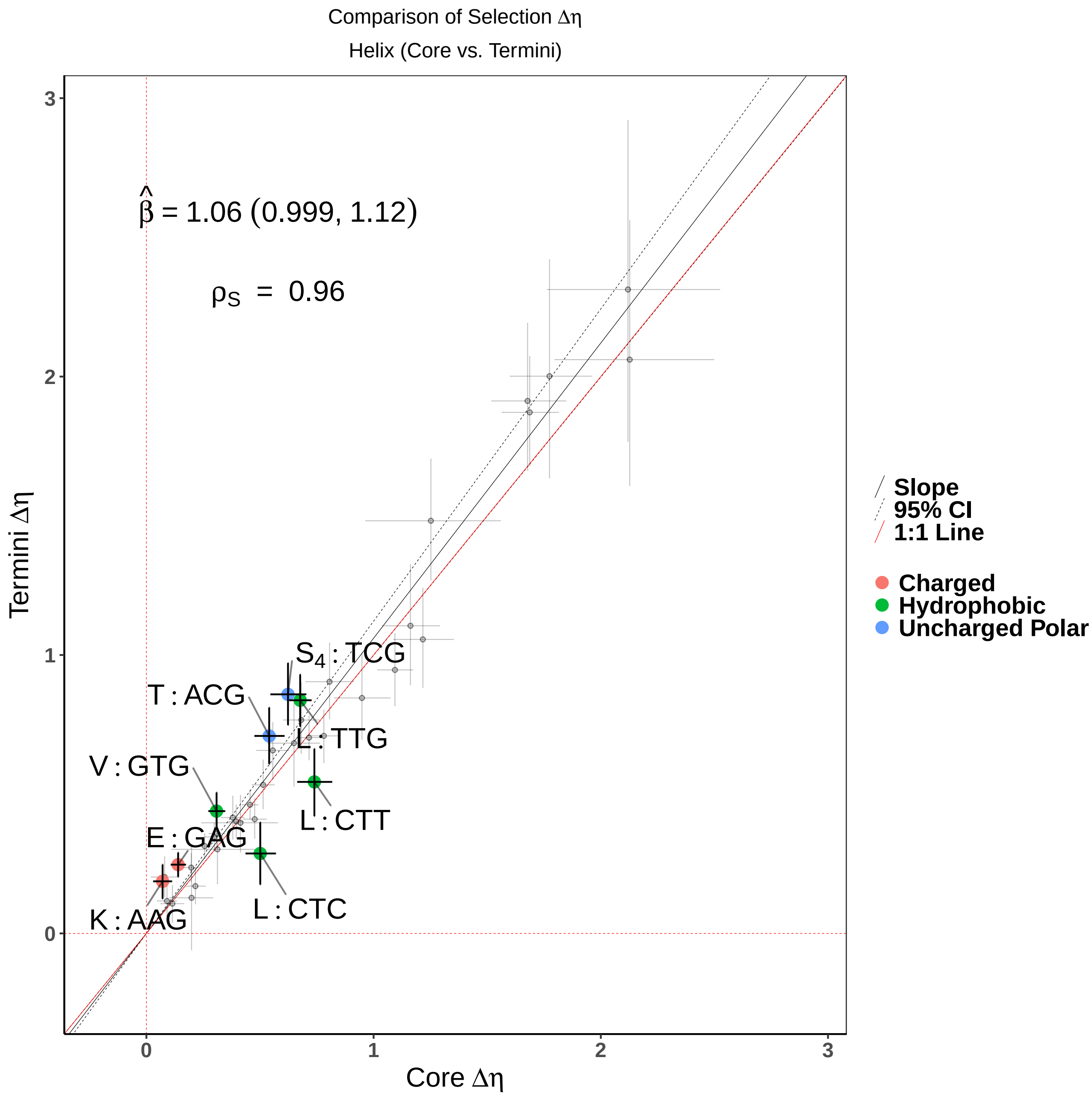

Figure S12: Comparison of selection $\Delta\eta$ between the termini and the core of helices in *E. coli*. Data was restricted to helices of minimum length 6 and treating the termini as the first and last 2 amino acids.

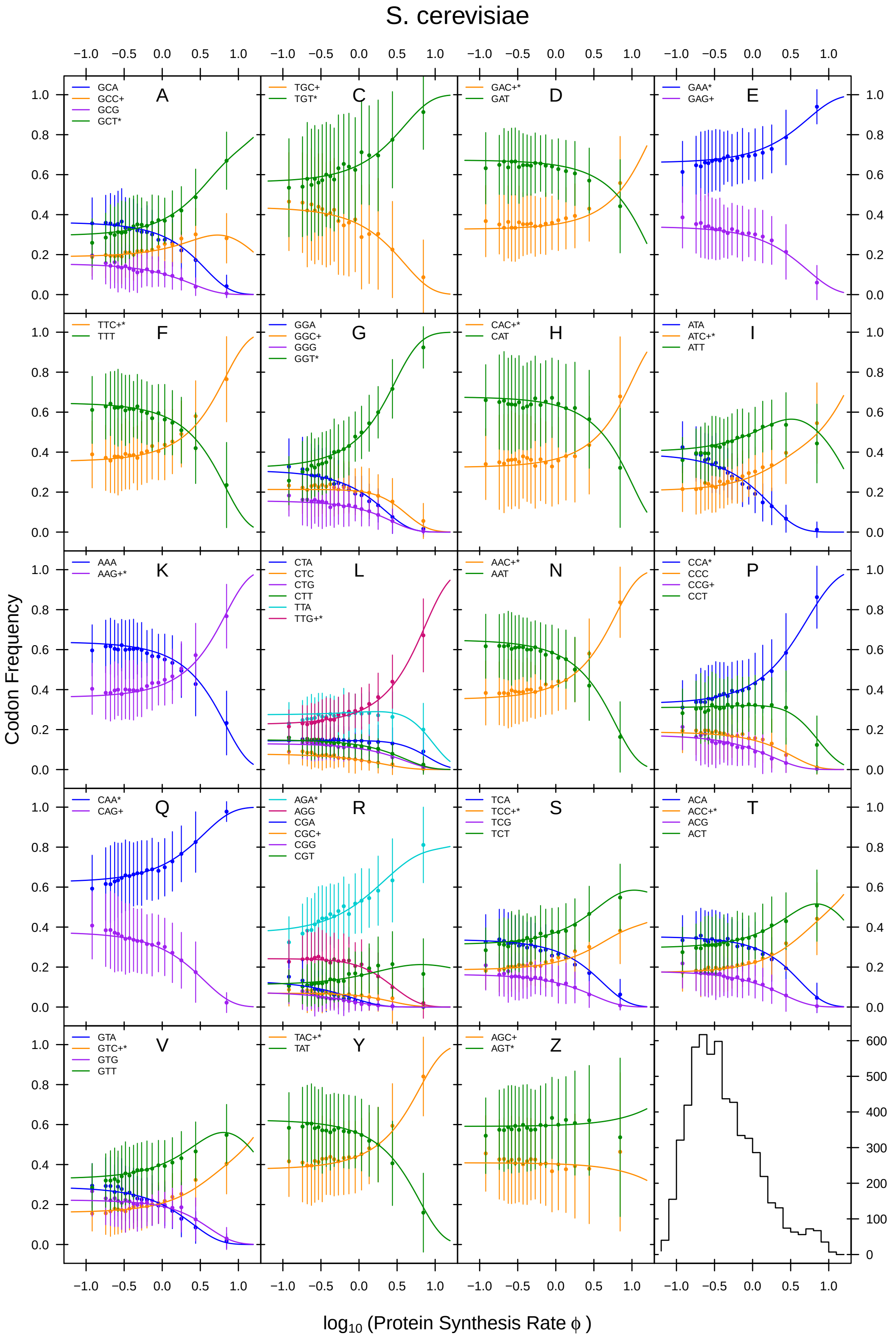

Figure S13: Observed (points) and expected (lines) codon frequencies for each amino acid as a function of protein synthesis (production) rates ϕ, as well as the distribution of ϕ for all genes. Note that serine is split into two amino acid groups, S = S_4 and Z = S_2. An * indicates the preferred codon identified by ROC-SEMPPR, while + indicates the preferred codon identified by nTE.

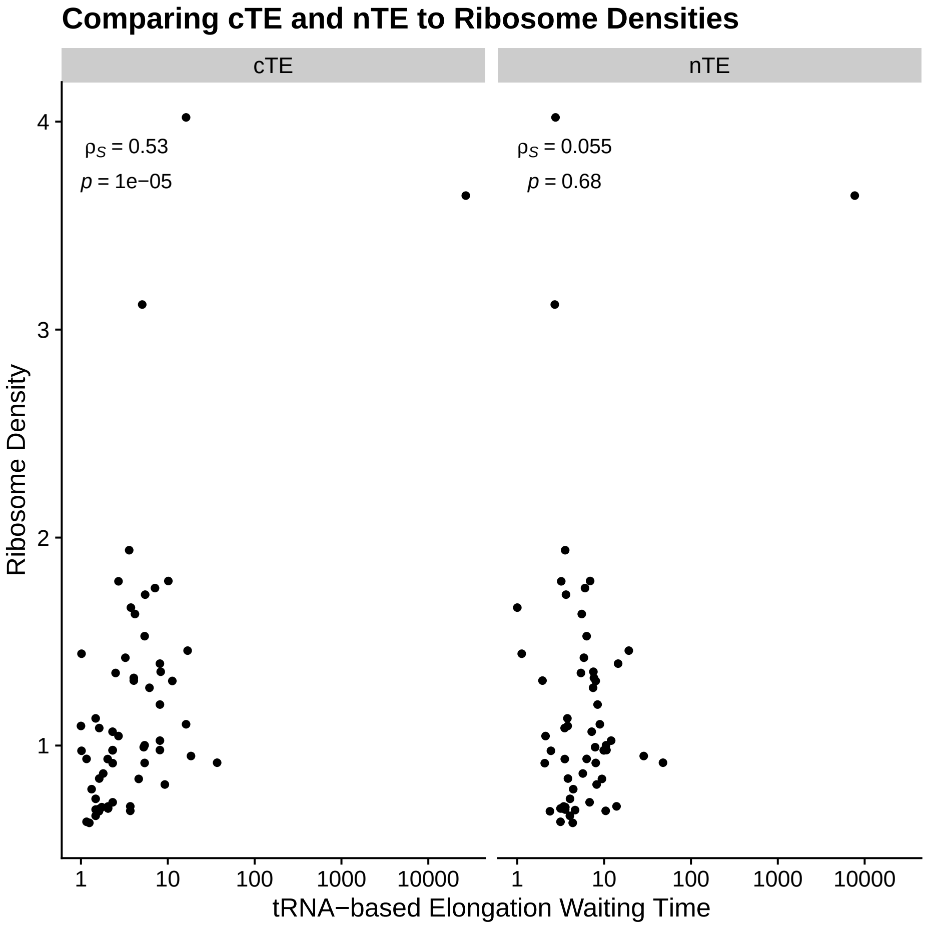

Figure S14: Comparing the classical translation efficiency metric (cTE, the same as the individual codon weights estimated in tAI) and normalized translation efficiency (nTE)[1] to ribosome densities estimated in [2]. As ribosome densities represent codon-specific elongation waiting times, we calculate tRNA-based elongation waiting times by calculating 1/cTE and 1/nTE. ρ_S represents the Spearman rank correlation coefficient.

Supplemental Tables

Table S1: Comparison of model fits examining variation in codon usage in *S. cerevisiae* categorized using empirically-determined protein secondary structures from PDB. Models with a DIC score greater than the null model (no difference in codon usage between secondary structures) were excluded from the table. H: helix. E: sheet. C: coil.

	Groupings			
Model	I	II	III	ΔDIC
Y_1	H	E	C	0.00
Y_2	HE	–	C	1
Y_4	HC	E	–	21
Y_0	HEC	–	–	34

Table S2: Comparison of model fits examining variation in codon usage in *E. coli* categorized using empirically-determined protein secondary structures from PDB. Models with a DIC score greater than the null model (no difference in codon usage between secondary structures) were excluded from the table. H: helix. E: sheet. C: coil.

	Groupings			
Model	I	II	III	ΔDIC
E_1	H	E	C	0.00
E_2	HE	–	C	20
E_3	H	EC	–	90
E_4	HC	E	–	101
E_0	HEC	–	–	145

Table S3: Breakdown of predictions by PsiPred (secondary structures) and IUPRED2 (disordered) in *S. cerevisiae*. Each value represents the number of amino acids falling into the corresponding categories. The percentages are relative to total number of amino acids predicted to be structured and disordered.

	Structured	Disordered
Coil	1,015,435 (0.43)	451,974 (0.79)
α-helix	1,062,128 (0.45)	106,645 (0.19)
β-sheet	281,425 (0.12)	13,899 (0.02)

Table S4: Comparing models for *S. cerevisiae* with termini of empirically-determined secondary structures separated from the core of the structure only considering secondary structures of 4 to 8 amino acids or longer and with the N and C-termini defined as the first and last 2 amino acids, respectively. H: helix. E: sheet. C: coil. (Next page)

S. cerervisiae with Termini Length: 2 amino acids

Minimum Structure Length	Model	Structure	Groupings I	Groupings II	Groupings III	ΔDIC
4	Y_{1b}	H	Termini	Core	–	0
	Y_{1a}		Whole Structure	–	–	14
	Y_{1c}		N-terminus	Core	C-terminus	20
	Y_{1d}	E	Whole Structure	–	–	0
	Y_{1e}		Termini	Core	–	39
	Y_{1f}		N-terminus	Core	C-terminus	75
	Y_{1g}	C	Whole Structure	–	–	0
	Y_{1h}		Termini	Core	–	38
	Y_{1i}		N-terminus	Core	C-terminus	82
5	Y_{1b}	H	Termini	Core	–	0
	Y_{1a}		Whole Structure	–	–	3
	Y_{1c}		N-terminus	Core	C-terminus	27
	Y_{1d}	E	Whole Structure	–	–	0
	Y_{1e}		Termini	Core	–	37
	Y_{1f}		N-terminus	Core	C-terminus	73
	Y_{1g}	C	Whole Structure	–	–	0
	Y_{1h}		Termini	Core	–	39
	Y_{1i}		N-terminus	Core	C-terminus	86
6	Y_{1a}	H	Whole Structure	–	–	0
	Y_{1b}		Termini	Core	–	2
	Y_{1c}		N-terminus	Core	C-terminus	33
	Y_{1d}	E	Whole Structure	–	–	0
	Y_{1e}		Termini	Core	–	33
	Y_{1f}		N-terminus	Core	C-terminus	57
	Y_{1g}	C	Whole Structure	–	–	0
	Y_{1h}		Termini	Core	–	47
	Y_{1i}		N-terminus	Core	C-terminus	96
7	Y_{1a}	H	Whole Structure	–	–	0
	Y_{1b}		Termini	Core	–	14
	Y_{1c}		N-terminus	Core	C-terminus	44
	Y_{1d}	E	Whole Structure	–	–	0
	Y_{1e}		Termini	Core	–	24
	Y_{1f}		N-terminus	Core	C-terminus	49
	Y_{1h}	C	Whole Structure	–	–	0
	Y_{1i}		Termini	Core	–	40
	Y_{1j}		N-terminus	Core	C-terminus	91
8	Y_{1a}	H	Whole Structure	–	–	0
	Y_{1b}		Termini	Core	–	6
	Y_{1c}		N-terminus	Core	C-terminus	38
	Y_{1d}	E	Whole Structure	–	–	0
	Y_{1e}		Termini	Core	–	37
	Y_{1f}		N-terminus	Core	C-terminus	57
	Y_{1g}	C	Whole Structure	–	–	0
	Y_{1h}		Termini	Core	–	36
	Y_{1i}		N-terminus	Core	C-terminus	85

Table S5: Comparing models for *S. cerevisiae* with termini of empirically-determined secondary structures separated from the core of the structure only considering secondary structures of 6 to 10 amino acids or longer and with the N and C-termini defined as the first and last 3 amino acids, respectively. H: helix. E: sheet. C: coil. (Next page)

Minimum Structure Length	Model	Structure	Groupings I	Groupings II	Groupings III	ΔDIC
6	Y_{1a}	H	Whole Structure	–	–	0
	Y_{1b}		Termini	Core	–	3
	Y_{1c}		N-terminus	Core	C-terminus	26
	Y_{1d}	E	Whole Structure	–	–	0
	Y_{1e}		Termini	Core	–	46
	Y_{1f}		N-terminus	Core	C-terminus	86
	Y_{1g}	C	Whole Structure	–	–	0
	Y_{1h}		Termini	Core	–	42
	Y_{1i}		N-terminus	Core	C-terminus	94
7	Y_{1a}	H	Whole Structure	–	–	0
	Y_{1b}		Termini	Core	–	7
	Y_{1c}		N-terminus	Core	C-terminus	33
	Y_{1d}	E	Whole Structure	–	–	0
	Y_{1e}		Termini	Core	–	45
	Y_{1f}		N-terminus	Core	C-terminus	88
	Y_{1g}	C	Whole Structure	–	–	0
	Y_{1h}		Termini	Core	–	49
	Y_{1i}		N-terminus	Core	C-terminus	103
8	Y_{1b}	H	Termini	Core	–	0
	Y_{1a}		Whole Structure	–	–	1
	Y_{1c}		N-terminus	Core	C-terminus	28
	Y_{1d}	E	Whole Structure	–	–	0
	Y_{1e}		Termini	Core	–	49
	Y_{1f}		N-terminus	Core	C-terminus	89
	Y_{1g}	C	Whole Structure	–	–	0
	Y_{1h}		Termini	Core	–	44
	Y_{1i}		N-terminus	Core	C-terminus	94
9	Y_{1b}	H	Termini	Core	–	0
	Y_{1a}		Whole Structure	–	–	3
	Y_{1c}		N-terminus	Core	C-terminus	40
	Y_{1d}	E	Whole Structure	–	–	0
	Y_{1e}		Termini	Core	–	46
	Y_{1f}		N-terminus	Core	C-terminus	86
	Y_{1g}	C	Whole Structure	–	–	0
	Y_{1h}		Termini	Core	–	36
	Y_{1i}		N-terminus	Core	C-terminus	79
10	Y_{1a}	H	Whole Structure	–	–	0
	Y_{1b}		Termini	Core	–	13
	Y_{1c}		N-terminus	Core	C-terminus	63
	Y_{1d}	E	Whole Structure	–	–	0
	Y_{1e}		Termini	Core	–	43
	Y_{1f}		N-terminus	Core	C-terminus	72
	Y_{1g}	C	Whole Structure	–	–	0
	Y_{1h}		Termini	Core	–	33
	Y_{1i}		N-terminus	Core	C-terminus	74

S. cerervisiae with Termini Length: 3 amino acids

Table S6: Effects of minimum α-helix (DSSP H) length and termini length for evaluating differences in selection at termini vs. the core of the structure. 3_10-helices (DSSP G) and π-helices (DSSP I) are excluded.

Minimum α-helix Length	Terminus Length	Category 1	Category 2	ΔDIC
4	2	Core	Termini	0
		Whole Structure	–	6
5	2	Core	Termini	0
		Whole Structure	–	2
6	2	Whole Structure		0
		Core	Termini	3
7	2	Whole Structure	–	0
		Core	Termini	21
8	2	Whole Structure	–	0
		Core	Termini	14
9	2	Whole Structure	–	0
		Core	Termini	13

Table S7: Comparing models for *E. coli* with termini of empirically-determined secondary structures separated from the core of the structure only considering secondary structures of 4 to 7 amino acids or longer and with the N and C-termini defined as the first and last 2 amino acids, respectively. H: helix. E: sheet. C: coil.

E. coli with Termini Length: 2 amino acids

Minimum Structure Length	Model	Structure	Groupings I	II	III	ΔDIC
4	E_{1b}	H	Termini	Core	–	0
	E_{1c}		N-terminus	Core	C-terminus	22
	E_{1a}		Whole Structure	–	–	91
	E_{1f}	E	N-terminus	Core	C-terminus	0
	E_{1d}		Whole Structure	–	–	48
	E_{1e}		Termini	Core	–	88
	E_{1g}	C	Whole Structure	–	–	0
	E_{1h}		Termini	Core	–	3
	E_{1i}		N-terminus	Core	C-terminus	8
5	E_{1b}	H	Termini	Core	–	0
	E_{1c}		N-terminus	Core	C-terminus	15
	E_{1a}		Whole Structure	–	–	75
	E_{1f}	E	N-terminus	Core	C-terminus	0
	E_{1d}		Whole Structure	–	–	24
	E_{1e}		Termini	Core	–	61
	E_{1g}	C	Whole Structure	–	–	0
	E_{1i}		N-terminus	Core	C-terminus	5
	E_{1h}		Termini	Core	–	8
6	E_{1b}	H	Termini	Core	–	0
	E_{1c}		N-terminus	Core	C-terminus	13
	E_{1a}		Whole Structure	–	–	64
	E_{1e}	E	Whole Structure	–	–	0
	E_{1g}		N-terminus	Core	C-terminus	37
	E_{1f}		Termini	Core	–	43
	E_{1h}	C	Whole Structure	–	–	0
	E_{1i}		Termini	Core	–	6
	E_{1j}		N-terminus	Core	C-terminus	14
7	E_{1b}	H	Termini	Core	–	0
	E_{1c}		N-terminus	Core	C-terminus	14
	E_{1a}		Whole Structure	–	–	44
	E_{1d}	E	Whole Structure	–	–	0
	E_{1e}		Termini	Core	–	49
	E_{1f}		N-terminus	Core	C-terminus	53
	E_{1g}	C	Whole Structure	–	–	0
	E_{1h}		Termini	Core	–	18
	E_{1i}		N-terminus	Core	C-terminus	45

References

[1] Pechmann S, Frydman J. Evolutionary conservation of codon optimality reveals hidden signatures of cotranslational folding. Nature Structural and Molecular Biology. 2013;20:237–243.

[2] Weinberg DE, Shah P, Eichhorn SW, Hussmann JA, Plotkin JB, Bartel DP. Improved Ribosome-Footprint and mRNA Measurements Provide Insights into Dynamics and Regulation of Yeast Translation. Cell Reports. 2016 2;14:1787–1799.
